# Supplementary material for: Cascaded dissipative DNAzyme-driven layered networks guide transient replication of coded-strands as gene models
Source: Nat Commun. 2022 Jul 29;13:4414. doi: 10.1038/s41467-022-32148-9 (PMC9338015; doi:10.1038/s41467-022-32148-9)
Supplement: Supplementary file 1 — Supplementary Information [file 41467_2022_32148_MOESM1_ESM.pdf]

Supplementary Information

Cascaded dissipative DNAzyme-driven layered networks guide transient replication of coded-strands as gene models

Jianbang Wang[1,2], Zhenzhen Li[1,2], Itamar Willner[1]*

[1] The Institute of Chemistry, The Center for Nanoscience and Nanotechnology,

The Hebrew University of Jerusalem, Jerusalem 91904, Israel.

[2] These authors contributed equally.

*Correspondence to: Itamar.willner@mail.huji.ac.il

Table of Contents

Supplementary Figures 1-60 2-72

Supplementary Tables 1-7 73-80

Supplementary References 81

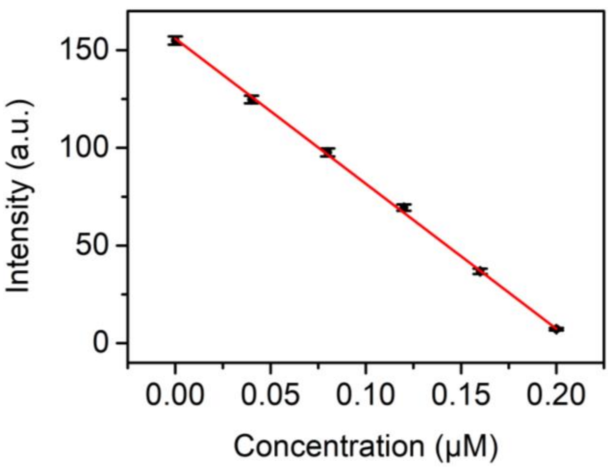

Supplementary Figure 1. Calibration curve corresponding to the fluorescence intensities of strand A (0.2 μM), in the presence of variable concentrations of the strand A'. The curve is fit linearly and the $r^2 = 0.99945$.

Kinetic equations of the dissipative module I shown in Figure 1:

$$AA' + T_1 \underset{k_{-1}}{\overset{k_1}{\rightleftharpoons}} T_1A + A' \quad (1)$$

$$T_1A + H_1 \underset{k_{-2}}{\overset{k_2}{\rightleftharpoons}} T_1AH_1 \quad (2)$$

$$T_1AH_1 \xrightarrow{k_3} T_1AH_{1a}H_{1b} \quad (3)$$

$$T_1AH_{1a}H_{1b} \underset{k_{-4}}{\overset{k_4}{\rightleftharpoons}} T_1A + H_{1a}H_{1b} \quad (4)$$

$$H_{1a}H_{1b} \underset{k_{-5}}{\overset{k_5}{\rightleftharpoons}} H_{1a} + H_{1b} \quad (5)$$

$$T_1A + H_{1a} \underset{k_{-6}}{\overset{k_6}{\rightleftharpoons}} T_1H_{1a} + A \quad (6)$$

$$A + A' \underset{k_{-7}}{\overset{k_7}{\rightleftharpoons}} AA' \quad (7)$$

Derivatives:

$$\frac{dAA'}{dt} = -k_1[AA'][T_1] + k_{-1}[T_1A][A'] + k_7[A][A'] - k_{-7}[AA']$$

$$\frac{dT_1}{dt} = -k_1[AA'][T_1] + k_{-1}[T_1A][A']$$

$$\frac{dT_1A}{dt} = k_1[AA'][T_1] - k_{-1}[T_1A][A'] - k_2[T_1A][H_1] + k_{-2}[T_1AH_1] + k_4[T_1AH_{1a}H_{1b}] - k_{-4}[T_1A][H_{1a}H_{1b}]\,] - k_6[T_1A][H_{1a}] + k_{-6}[T_1H_{1a}][A]$$

$$\frac{dA'}{dt} = k_1[AA'][T_1] - k_{-1}[T_1A][A'] - k_7[A][A'] + k_{-7}[AA']$$

$$\frac{dH_1}{dt} = -k_2[T_1A][H_1] + k_{-2}[T_1AH_1]$$

$$\frac{dT_1AH_1}{dt} = k_2[T_1A][H_1] - k_{-2}[T_1AH_1] - k_3[T_1AH_1]$$

$$\frac{dT_1AH_{1a}H_{1b}}{dt} = k_3[T_1AH_1] - k_4[T_1AH_{1a}H_{1b}] + k_{-4}[T_1A][H_{1a}H_{1b}]$$

$$\frac{dH_{1a}H_{1b}}{dt} = k_4[T_1AH_{1a}H_{1b}] - k_{-4}[T_1A][H_{1a}H_{1b}] - k_5[H_{1a}H_{1b}] + k_{-5}[H_{1a}][H_{1b}]$$

$$\frac{dH_{1a}}{dt} = k_5[H_{1a}H_{1b}] - k_{-5}[H_{1a}][H_{1b}] - k_6[T_1A][H_{1a}] + k_{-6}[T_1H_{1a}][A]$$

$$\frac{dH_{1b}}{dt} = k_5[H_{1a}H_{1b}] - k_{-5}[H_{1a}][H_{1b}]$$

$$\frac{dT_1H_{1a}}{dt} = k_6[T_1A][H_{1a}] - k_{-6}[T_1H_{1a}][A]$$

$$\frac{dA}{dt} = k_6[T_1A][H_{1a}] - k_{-6}[T_1H_{1a}][A] - k_7[A][A'] + k_{-7}[AA']$$

Supplementary Figure 2. Computational simulation of the dissipative module I shown in Figure 1. The kinetic scheme of the reactions associated with the time-dependent concentration changes during the dissipative transitions of Mg^{2+}-ion-dependent DNAzyme T_1/A is summarized in above equations. Knowing the time-dependent concentration changes of the free unquenched fluorophore Cy5, we computationally simulated the time-dependent concentration changes by using Matlab R2020a.

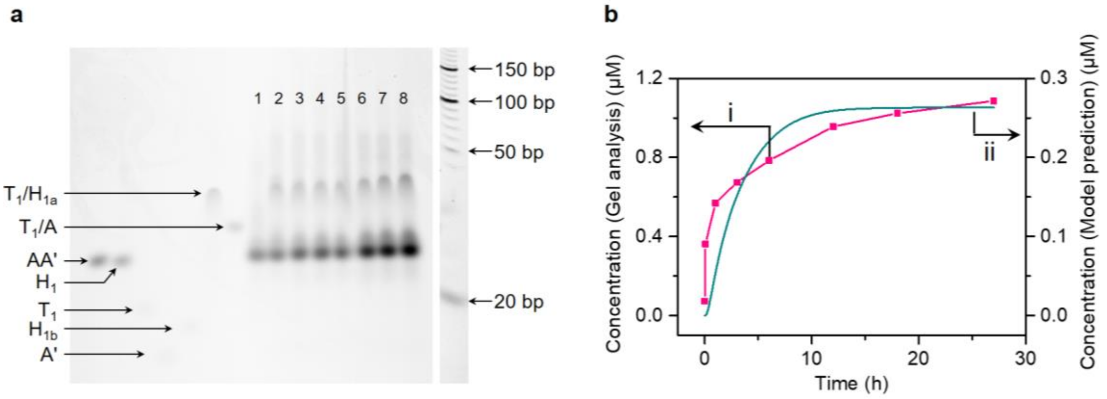

Supplementary Figure 3. Gel analysis of the transient dynamic changes of the T_1/H_{1a} of the dissipative module I shown in Figure 1. a, Image of the electrophoretic gel (10% PAGE, 80 V, 4 °C) corresponding to samples withdrawn at variable time-intervals from the dissipative module I shown in Figure 1. The lanes of 1-8 on the image correspond to the dissipative system at different time-intervals of 0, 0.05, 1, 3, 6, 12, 18 and 27 h, respectively. The separated bands were stained with dye Gel Red and quantitatively analyzed using ImageJ software (version 1.8.0). A reference ladder consisting of variable compositions of nucleic acids separated electrophoretically at similar conditions, is provided (right). A reference band of T_1/H_{1a} at a concentration of 0.8 μM was separated, and it allowed the quantitative evaluation of the transient constituent T_1/H_{1a} present in the system at time-intervals of operating the system. Obviously, the transient intensity changes of the band associated with T_1/H_{1a} represent the dynamics and transient formation of the waste product associated with the dynamic reaction module presented in Fig. 1a. In fact, this is the only clearly separable band from all the constituents participating in the transient operation of the module. Upon evaluation of the electrophoretic bands associated with the dynamic transient system, several comments may be mentioned: (i) The bands in lanes 2-8 reveal, as expected, a time-dependent increase in the contents of T_1/H_{1a} as the dynamic process proceeds. This is consistent with the continuous formation of the T_1/H_{1a} waste product. (ii) The bands associated with the waste product reveal a slightly, yet observable, slower migration rate, as compared to the reference migration rate of the reference T_1/H_{1a}, as the dynamic process proceeds. This might be attributed to accompanying dynamic aggregation of T_1/H_{1a}, being enhanced as the concentration of the waste product increase (realizing that the electrophoretic separation is conducted at 4 °C). (iii) The bands associated with AA' and H_1 reveal, however, increased time-dependent intensities and slightly slower migration rates, as the dynamic process proceeds. Although, we do not have a firm explanation to these intensified bands response, we speculate that the generated waste products H_{1b} and H_{1a} might partially dimerize as an intermediate (at 4 °C?) that is integrated in the band comprising of AA' and H_1. (iv) We note, however, the bands of T_1/H_{1a} reveal a transient time-dependent increase in content (Supplementary Fig. 3**b**) that is comparable to the computed time-dependent accumulation of the waste T_1/H_{1a} (Supplementary Fig. 3**b**, curves (i) and (ii)). **b**, Transient concentrations of T_1/H_{1a} provided by the electrophoretic separated bands (i) and transient predicted

concentration of T_1/H_{1a}, composition of the reaction corresponded to 0.2 μM AA', 1 μM H_1 and 0.3 μM T_1 (ii). Note that the initial concentrations of the constituents in the reaction module for the electrophoretic separation of the transient T_1/H_{1a} are higher than the concentrations used for the transient fluorescence characterization of the system. This is required to image the respective transient electrophoretically separated constituents.

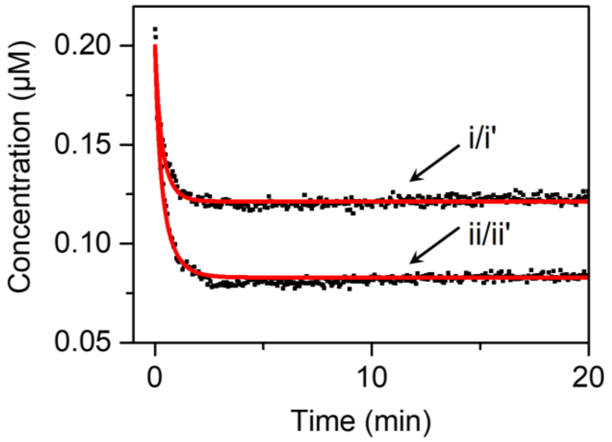

Supplementary Figure 4. Time-dependent concentration changes of strand A upon subjecting different concentrations of A': 0.08 μM (i/i') and 0.12 μM (ii/ii'). Solid curves correspond to the computationally simulated kinetic profiles. Dotted curves represent the experimental data.

Evaluation of the rate constants k_7 and k_{-7} of the kinetic scheme in Supplementary Figure 2.

To evaluate the rate constants k_7 and k_{-7} appearing in the kinetic scheme, equation (7) in Supplementary Figure 2, different concentrations of strand A' (0.08 μM and 0.12 μM) were subjected into the solutions of strand A (0.2 μM), and the time-dependent concentration changes were evaluated by following the fluorescence changes of the Cy5 that were translated into concentrations by applying an appropriate calibration curve, Supplementary Figure 1. From the kinetic profiles and using the Matlab R2020a program, the respective k_7 = 17.815 μM^{-1} min^{-1} and k_{-7} = 0.0367 min^{-1} were derived.

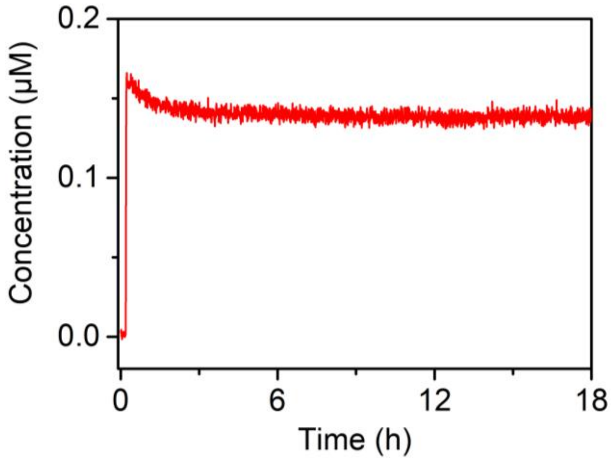

Supplementary Figure 5. Control experiment applying a hairpin H_{1c} that lacks the ribonucleobase in the loop domain. In the presence of the trigger T_1 (0.4 μM) only the displacement of the duplex A/A' occurs, without the dissipative recovery of the reaction module, implying that the cleavage of H_1 by DNAzyme T_1/A is essential to operate the transient system.

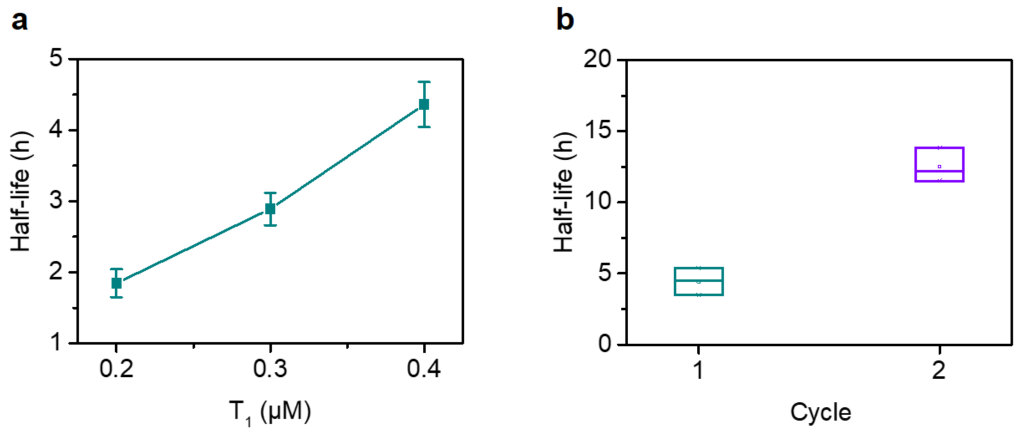

Supplementary Figure 6. Half-life of the DNAzyme T_1/A of module I. a, Half-life of the DNAzyme T_1/A of module I at variable concentrations of the trigger T_1. **b,** Half-life of the DNAzyme T_1/A of module I in the first and the second cycle showing in Figure 1**c**. Error bars were derived from N = 3 experiments.

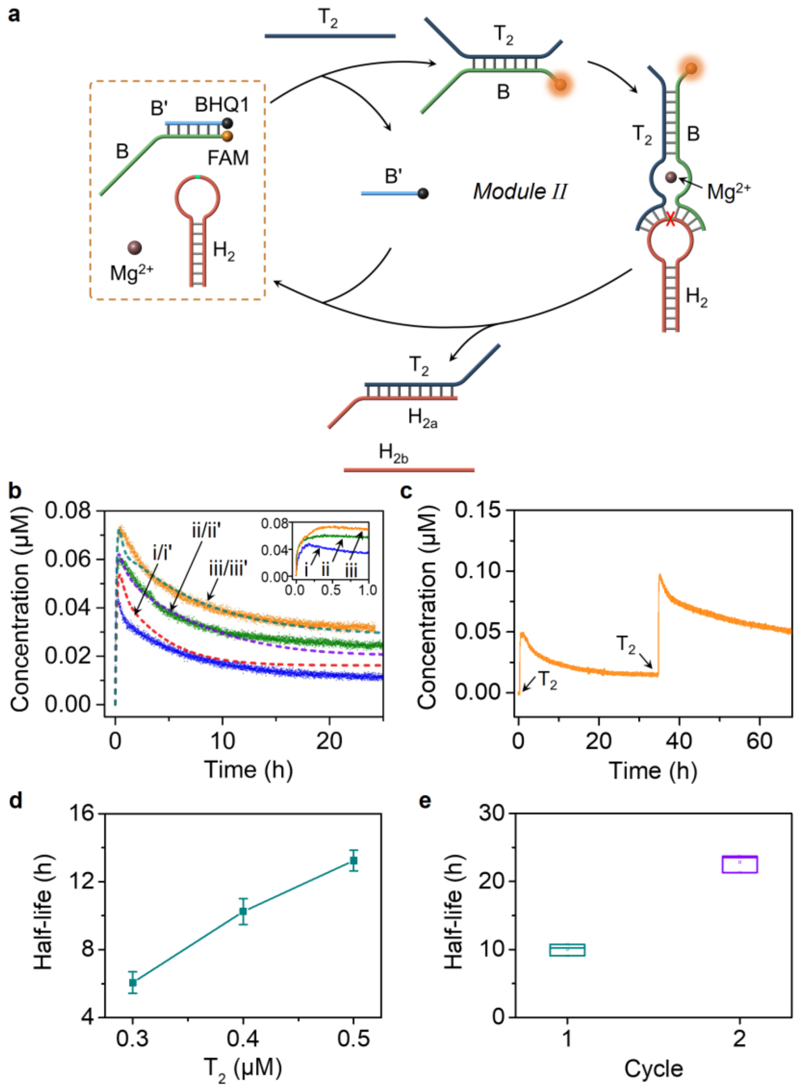

Supplementary Figure 7. Triggered transient formation and depletion of DNAzyme T_2/B. a, Triggered transient operation of the reaction module II results in the dissipative formation and depletion of a Mg^{2+}-ion-dependent DNAzyme T_2/B. **b**, Transient concentration profiles corresponding to the formation and depletion of the Mg^{2+}-ion-dependent DNAzyme T_2/B. Solid lines correspond to the experimental data generated in the presence of different concentrations of trigger (B/B' 0.2 μM, H_2 1 μM): (i) T_2 = 0.3 μM, (ii) T_2 = 0.4 μM and (iii) T_2 = 0.5 μM. Dashed lines correspond to the computationally simulated transient using the kinetic model presented in Supplementary Figure 9: (i') T_2 = 0.3 μM, (ii') T_2 = 0.4 μM and (iii') T_2 = 0.5 μM. Insert: The time-dependent dynamic evolution of T_2/B at short time intervals: (i) in the presence of trigger T_2 = 0.3 μM, (ii) in the presence of trigger T_2 = 0.4 μM, (iii) in the presence of trigger T_2 = 0.5 μM. **c**, Cyclic operation of the transient dissipative cycle (T_2 = 0.4 μM) of module II. Arrows indicate the addition of the trigger T_2. **d**, Half-life of the DNAzyme T_2/B of module II at variable concentrations of the trigger T_2. **e**, Half-life of the DNAzyme T_2/B of module II in the first and the second cycle showing in **c.** Error bars were derived from N = 3 experiments.

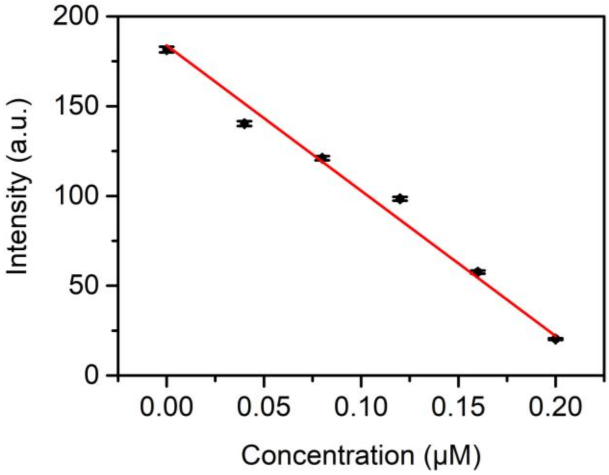

Supplementary Figure 8. Calibration curve corresponding to the fluorescence intensities of strand B (0.2 μM), in the presence of variable concentrations of the strand B'. The curve is fit linearly and the r^2 = 0.98715.

Kinetic equations of the dissipative module II shown in Supplementary Figure 7:

$$BB' + T_2 \underset{k_{-8}}{\overset{k_8}{\rightleftharpoons}} T_2B + B' \quad (8)$$

$$T_2B + H_2 \underset{k_{-9}}{\overset{k_9}{\rightleftharpoons}} T_2BH_2 \quad (9)$$

$$T_2BH_2 \xrightarrow{k_{10}} T_2BH_{2a}H_{2b} \quad (10)$$

$$T_2BH_{2a}H_{2b} \underset{k_{-11}}{\overset{k_{11}}{\rightleftharpoons}} T_2B + H_{2a}H_{2b} \quad (11)$$

$$H_{2a}H_{2b} \underset{k_{-12}}{\overset{k_{12}}{\rightleftharpoons}} H_{2a} + H_{2b} \quad (12)$$

$$T_2B + H_{2a} \underset{k_{-13}}{\overset{k_{13}}{\rightleftharpoons}} T_2H_{2a} + B \quad (13)$$

$$B + B' \underset{k_{-14}}{\overset{k_{14}}{\rightleftharpoons}} BB' \quad (14)$$

Derivatives:

$$\frac{dBB'}{dt} = -k_8[BB'][T_2] + k_{-8}[T_2B][B'] + k_{14}[B][B'] - k_{-14}[BB']$$

$$\frac{dT_2}{dt} = -k_8[BB'][T_2] + k_{-8}[T_2B][B']$$

$$\frac{dT_2B}{dt} = k_8[BB'][T_2] - k_{-8}[T_2B][B'] - k_9[T_2B][H_2] + k_{-9}[T_2BH_2] + k_{11}[T_2BH_{2a}H_{2b}] - k_{-11}[T_2B][H_{2a}H_{2b}]\ ] - k_{13}[T_2B][H_{2a}] + k_{-13}[T_2H_{2a}][B]$$

$$\frac{dB'}{dt} = k_8[BB'][T_2] - k_{-8}[T_2B][B'] - k_{14}[B][B'] + k_{-14}[BB']$$

$$\frac{dH_2}{dt} = -k_9[T_2B][H_2] + k_{-9}[T_2BH_2]$$

$$\frac{dT_2BH_2}{dt} = k_9[T_2B][H_2] - k_{-9}[T_2BH_2] - k_{10}[T_2BH_2]$$

$$\frac{dT_2BH_{2a}H_{2b}}{dt} = k_{10}[T_2BH_2] - k_{11}[T_2BH_{2a}H_{2b}] + k_{-11}[T_2B][H_{2a}H_{2b}]$$

$$\frac{dH_{2a}H_{2b}}{dt} = k_{11}[T_2BH_{2a}H_{2b}] - k_{-11}[T_2B][H_{2a}H_{2b}] - k_{12}[H_{2a}H_{2b}] + k_{-12}[H_{2a}][H_{2b}]$$

$$\frac{dH_{2a}}{dt} = k_{12}[H_{2a}H_{2b}] - k_{-12}[H_{2a}][H_{2b}] - k_{13}[T_2B][H_{2a}] + k_{-13}[T_2H_{2a}][B]$$

$$\frac{dH_{2b}}{dt} = k_{12}[H_{2a}H_{2b}] - k_{-12}[H_{2a}][H_{2b}]$$

$$\frac{dT_2H_{2a}}{dt} = k_{13}[T_2B][H_{2a}] - k_{-13}[T_2H_{2a}][B]$$

$$\frac{dB}{dt} = k_{13}[T_2B][H_{2a}] - k_{-13}[T_2H_{2a}][B] - k_{14}[B][B'] + k_{-14}[BB']$$

Supplementary Figure 9. Computational simulation of the dissipative module II shown in Supplementary Figure 7. The kinetic scheme of the reactions associated with the time-dependent concentration changes during the dissipative transitions of Mg^{2+}-ion-dependent DNAzyme T_2/B is summarized in above equations. Knowing the time-dependent concentration changes of the free unquenched fluorophore FAM, we computationally simulated the time-dependent concentration changes by using Matlab R2020a.

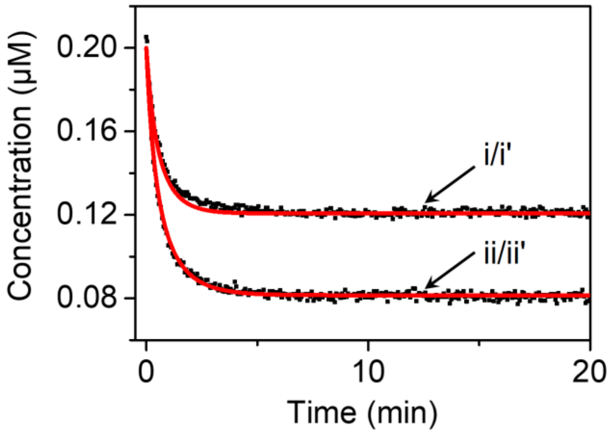

Supplementary Figure 10. Time-dependent concentration changes of strand B upon subjecting different concentrations of B': 0.08 μM (i/i') and 0.12 μM (ii/ii'). Solid curves correspond to the computationally simulated kinetic profiles. Dotted curves represent the experimental data.

Evaluation of the rate constants k_{14} and k_{-14} of the kinetic scheme in Supplementary Figure 9.

To evaluate the rate constants k_{14} and k_{-14} appearing in the kinetic scheme, equation (14) in Supplementary Figure 9, different concentrations of strand B' (0.08 μM and 0.12 μM) were subjected into the solutions of strand B (0.2 μM), and the time-dependent concentration changes were evaluated by following the fluorescence changes of the FAM that were translated into concentrations by applying an appropriate calibration curve, Supplementary Figure 8. From the kinetic profiles and using the Matlab R2020a program, the respective k_{14} = 10.3 μM^{-1} min^{-1} and k_{-14} = 0.01 min^{-1} were derived.

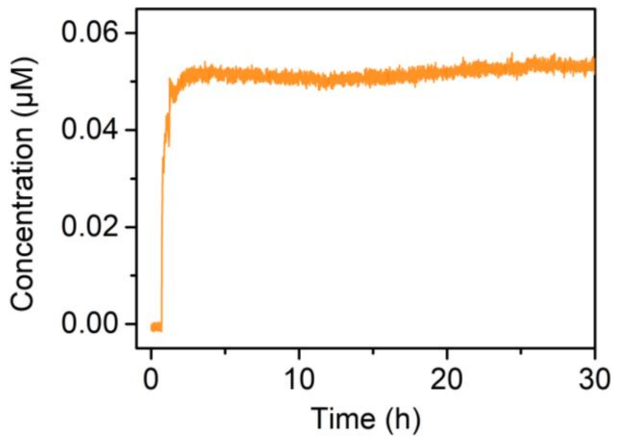

Supplementary Figure 11. Control experiment applying a hairpin H_{2c} that lacks the ribonucleobase in the loop domain. In the presence of the trigger T_2 (0.4 µM) only the displacement of the duplex B/B' occurs, without the dissipative recovery of the reaction module II, implying that the cleavage of H_2 by DNAzyme T_2/B is essential to operate the transient system.

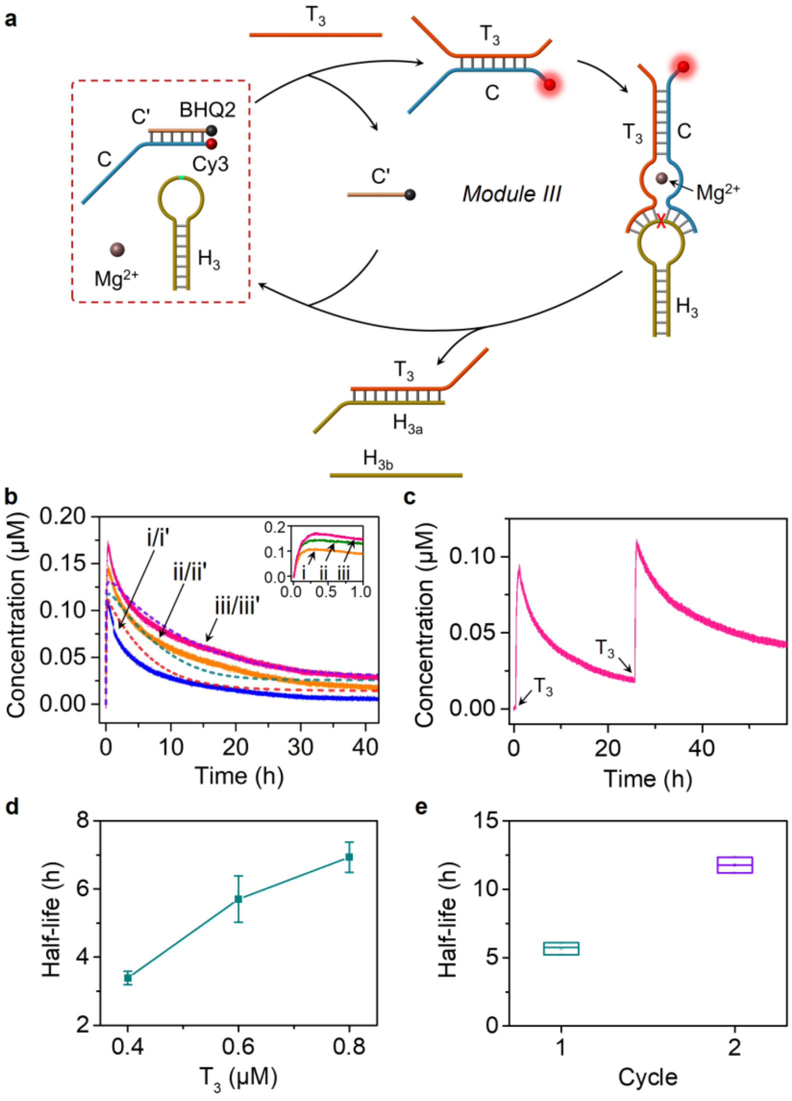

Supplementary Figure 12. Triggered transient formation and depletion of DNAzyme T_3/C. a, Triggered transient operation of the reaction module III results in the dissipative formation and depletion of a Mg^{2+}-ion-dependent DNAzyme T_3/C. **b**, Transient concentration profiles corresponding to the formation and depletion of the Mg^{2+}-ion-dependent DNAzyme T_3/C. Solid lines correspond to the experimental data generated in the presence of different concentrations of trigger (C/C' 0.2 μM, H_3 1 μM): (i) T_3 = 0.4 μM, (ii) T_3 = 0.6 μM and (iii) T_3 = 0.8 μM. Dashed lines correspond to the computationally simulated transient using the kinetic model presented in Supplementary Figure 14: (i') T_3 = 0.4 μM, (ii') T_3 = 0.6 μM and (iii') T_3 = 0.8 μM. Insert: The time-dependent dynamic evolution of T_3/C at short time intervals: (i) in the presence of trigger T_3 = 0.4 μM, (ii) in the presence of trigger T_3 = 0.6 μM, (iii) in the presence of trigger T_3 = 0.8 μM. **c**, Cyclic operation of the transient dissipative cycle (T_3 = 0.4 μM) of module III. Arrows indicate the addition of the trigger T_3. **d**, Half-life of the DNAzyme T_3/C of module III at variable concentrations of the trigger T_3. **e**, Half-life of the DNAzyme T_3/C of module III in the first and the second cycle showing in **c.** Error bars were derived from N = 3 experiments.

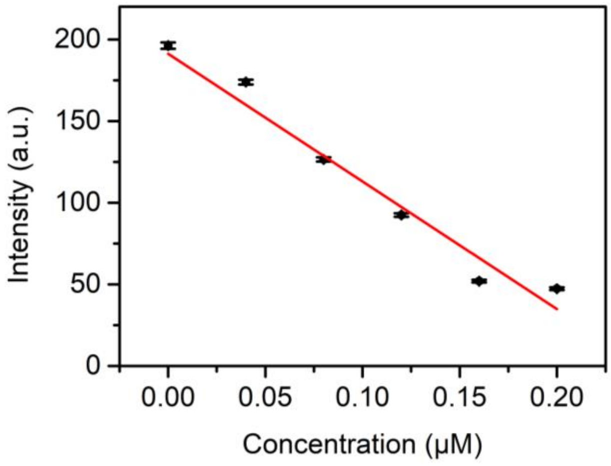

Supplementary Figure 13. Calibration curve corresponding to the fluorescence intensities of strand C (0.2 µM), in the presence of variable concentrations of the strand C'. The curve is fit linearly and the r^2 = 0.93226.

Kinetic equations of the dissipative module III shown in Supplementary Figure 12:

$$CC' + T_3 \underset{k_{-15}}{\overset{k_{15}}{\rightleftharpoons}} T_3C + C' \qquad (15)$$

$$T_3C + H_3 \underset{k_{-16}}{\overset{k_{16}}{\rightleftharpoons}} T_3CH_3 \qquad (16)$$

$$T_3CH_3 \xrightarrow{k_{17}} T_3CH_{3a}H_{3b} \qquad (17)$$

$$T_3CH_{3a}H_{3b} \underset{k_{-18}}{\overset{k_{18}}{\rightleftharpoons}} T_3C + H_{3a}H_{3b} \qquad (18)$$

$$H_{3a}H_{3b} \underset{k_{-19}}{\overset{k_{19}}{\rightleftharpoons}} H_{3a} + H_{3b} \qquad (19)$$

$$T_3C + H_{3a} \underset{k_{-20}}{\overset{k_{20}}{\rightleftharpoons}} T_3H_{3a} + C \qquad (20)$$

$$C + C' \underset{k_{-21}}{\overset{k_{21}}{\rightleftharpoons}} CC' \qquad (21)$$

Derivatives:

$$\frac{dCC'}{dt} = -k_{15}[CC'][T_3] + k_{-15}[T_3C][C'] + k_{21}[C][C'] - k_{-21}[CC']$$

$$\frac{dT_3}{dt} = -k_{15}[CC'][T_3] + k_{-15}[T_3C][C']$$

$$\frac{dT_3C}{dt} = k_{15}[CC'][T_3] - k_{-15}[T_3C][C'] - k_{16}[T_3C][H_3] + k_{-16}[T_3CH_3] + k_{18}[T_3CH_{3a}H_{3b}] - k_{-18}[T_3C][H_{3a}H_{3b}]\,] - k_{20}[T_3C][H_{3a}] + k_{-20}[T_3H_{3a}][C]$$

$$\frac{dC'}{dt} = k_{15}[CC'][T_3] - k_{-15}[T_3C][C'] - k_{21}[C][C'] + k_{-21}[CC']$$

$$\frac{dH_3}{dt} = -k_{16}[T_3C][H_3] + k_{-16}[T_3CH_3]$$

$$\frac{dT_3CH_3}{dt} = k_{16}[T_3C][H_3] - k_{-16}[T_3CH_3] - k_{17}[T_3CH_3]$$

$$\frac{dT_3CH_{3a}H_{3b}}{dt} = k_{17}[T_3CH_3] - k_{18}[T_3CH_{3a}H_{3b}] + k_{-18}[T_3C][H_{3a}H_{3b}]$$

$$\frac{dH_{3a}H_{3b}}{dt} = k_{18}[T_3CH_{3a}H_{3b}] - k_{-18}[T_3\,C][H_{3a}H_{3b}] - k_{19}[H_{3a}H_{3b}] + k_{-19}[H_{3a}][H_{3b}]$$

$$\frac{dH_{3a}}{dt} = k_{19}[H_{3a}H_{3b}] - k_{-19}[H_{3a}][H_{3b}] - k_{20}[T_3C][H_{3a}] + k_{-20}[T_3H_{3a}][C]$$

$$\frac{dH_{3b}}{dt} = k_{19}[H_{3a}H_{3b}] - k_{-19}[H_{3a}][H_{3b}]$$

$$\frac{dT_3H_{3a}}{dt} = k_{20}[T_3C][H_{3a}] - k_{-20}[T_3H_{3a}][C]$$

$$\frac{dC}{dt} = k_{20}[T_3C][H_{3a}] - k_{-20}[T_3H_{3a}][C] - k_{21}[C][C'] + k_{-21}[CC']$$

Supplementary Figure 14. Computational simulation of the dissipative module III shown in Supplementary Figure 12. The kinetic scheme of the reactions associated with the time-dependent concentration changes during the dissipative transitions of Mg^{2+}-ion-dependent DNAzyme T_3/C is summarized in above equations. Knowing the time-dependent concentration changes of the free unquenched fluorophore Cy3, we computationally simulated the time-dependent concentration changes by using Matlab R2020a.

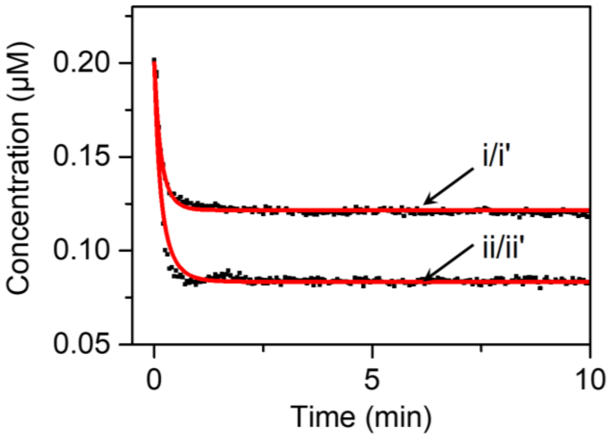

Supplementary Figure 15. Time-dependent concentration changes of strand C upon subjecting different concentrations of C': 0.08 µM (i/i') and 0.12 µM (ii/ii'). Solid curves correspond to the computationally simulated kinetic profiles. Dotted curves represent the experimental data.

Evaluation of the rate constants k_{21} and k_{-21} of the kinetic scheme in Supplementary Figure 14.

To evaluate the rate constants k_{21} and k_{-21} appearing in the kinetic scheme, equation (21) in Supplementary Figure 14, different concentrations of strand C' (0.08 µM and 0.12 µM) were subjected into the solutions of strand C (0.2 µM), and the time-dependent concentration changes were evaluated by the following fluorescence changes of the Cy3 that were translated into concentrations by applying an appropriate calibration curve, Supplementary Figure 13. From the kinetic profiles and using the Matlab R2020a program, the respective k_{21} = 37.477 μM^{-1} min^{-1} and k_{-21} = 0.0936 min^{-1} were derived.

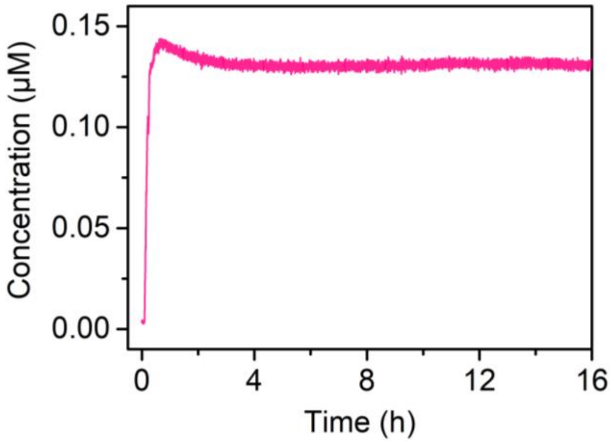

Supplementary Figure 16. Control experiment applying a hairpin H_{3c} that lacks the ribonucleobase in the loop domain. In the presence of the trigger T_3 (0.6 µM) only the displacement of the duplex C/C' occurs, without the dissipative recovery of the reaction module III, implying that the cleavage of H_3 by DNAzyme T_3/C is essential to operate the transient system.

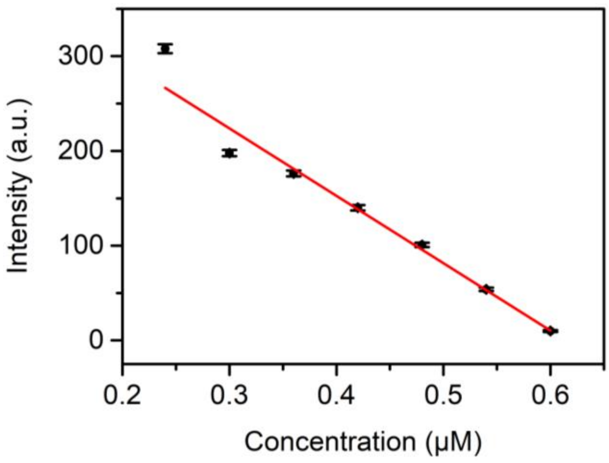

Supplementary Figure 17. Calibration curve corresponding to the fluorescence intensities of strand A (0.6 μM), in the presence of variable concentrations of the A'. The curve is fit linearly and the r^2 = 0.98154.

Kinetic equations of the dissipative cascaded bilayer module I and module II shown in Figure 2:

$$AA' + T_1 \underset{k_{-22}}{\overset{k_{22}}{\rightleftharpoons}} T_1A + A' \quad (22)$$

$$T_1A + H_1 \underset{k_{-23}}{\overset{k_{23}}{\rightleftharpoons}} T_1AH_1 \quad (23)$$

$$T_1AH_1 \xrightarrow{k_{24}} T_1AH_{1a}H_{1b} \quad (24)$$

$$T_1AH_{1a}H_{1b} \underset{k_{-25}}{\overset{k_{25}}{\rightleftharpoons}} T_1A + H_{1a}H_{1b} \quad (25)$$

$$H_{1a}H_{1b} \underset{k_{-26}}{\overset{k_{26}}{\rightleftharpoons}} H_{1a} + H_{1b} \quad (26)$$

$$T_1A + H_{1a} \underset{k_{-27}}{\overset{k_{27}}{\rightleftharpoons}} T_1H_{1a} + A \quad (27)$$

$$A + A' \underset{k_{-28}}{\overset{k_{28}}{\rightleftharpoons}} AA' \quad (28)$$

$$H_{1b} + T_2S \underset{k_{-36}}{\overset{k_{36}}{\rightleftharpoons}} T_2 + H_{1b}S \quad (36)$$

$$BB' + T_2 \underset{k_{-29}}{\overset{k_{29}}{\rightleftharpoons}} T_2B + B' \quad (29)$$

$$T_2B + H_2 \underset{k_{-30}}{\overset{k_{30}}{\rightleftharpoons}} T_2BH_2 \quad (30)$$

$$T_2BH_2 \xrightarrow{k_{31}} T_2BH_{2a}H_{2b} \quad (31)$$

$$T_2BH_{2a}H_{2b} \underset{k_{-32}}{\overset{k_{32}}{\rightleftharpoons}} T_2B + H_{2a}H_{2b} \quad (32)$$

$$H_{2a}H_{2b} \underset{k_{-33}}{\overset{k_{33}}{\rightleftharpoons}} H_{2a} + H_{2b} \quad (33)$$

$$T_2B + H_{2a} \underset{k_{-34}}{\overset{k_{34}}{\rightleftharpoons}} T_2H_{2a} + B \quad (34)$$

$$B + B' \underset{k_{-35}}{\overset{k_{35}}{\rightleftharpoons}} BB' \quad (35)$$

Derivatives:

$$\frac{dAA'}{dt} = -k_{22}[AA'][T_1] + k_{-22}[T_1A][A'] + k_{28}[A][A'] - k_{-28}[AA']$$

$$\frac{dT_1}{dt} = -k_{22}[AA'][T_1] + k_{-22}[T_1A][A']$$

$$\frac{dT_1A}{dt} = k_{22}[AA'][T_1] - k_{-22}[T_1A][A'] - k_{23}[T_1A][H_1] + k_{-23}[T_1AH_1] + k_{25}[T_1AH_{1a}H_{1b}] - k_{-25}[T_1A][H_{1a}H_{1b}]\,] - k_{27}[T_1A][H_{1a}] + k_{-27}[T_1H_{1a}][A]$$

$$\frac{dA'}{dt} = k_{22}[AA'][T_1] - k_{-22}[T_1A][A'] - k_{28}[A][A'] + k_{-28}[AA']$$

$$\frac{dH_1}{dt} = -k_{23}[T_1A][H_1] + k_{-23}[T_1AH_1]$$

$$\frac{dT_1AH_1}{dt} = k_{23}[T_1A][H_1] - k_{-23}[T_1AH_1] - k_{24}[T_1AH_1]$$

$$\frac{dT_1AH_{1aH1b}}{dt} = k_{24}[T_1AH_1] - k_{25}[T_1AH_{1a}H_{1b}] + k_{-25}[T_1A][H_{1a}H_{1b}]$$

$$\frac{dH_{1aH1b}}{dt} = k_{25}[T_1AH_{1a}H_{1b}] - k_{-25}[T_1A][H_{1a}H_{1b}] - k_{26}[H_{1a}H_{1b}] + k_{-26}[H_{1a}][H_{1b}]$$

$$\frac{dH_{1a}}{dt} = k_{26}[H_{1a}H_{1b}] - k_{-26}[H_{1a}][H_{1b}] - k_{27}[T_1A][H_{1a}] + k_{-27}[T_1H_{1a}][A]$$

$$\frac{dH_{1b}}{dt} = k_{26}[H_{1a}H_{1b}] - k_{-26}[H_{1a}][H_{1b}] - k_{36}[H_{1b}]\,[T_2S] + k_{-36}[T_2][H_{1b}S]$$

$$\frac{dT_1H_{1a}}{dt} = k_{27}[T_1A][H_{1a}] - k_{-27}[T_1H_{1a}][A]$$

$$\frac{dA}{dt} = k_{27}[T_1A][H_{1a}] - k_{-27}[T_1H_{1a}][A] - k_{28}[A][A'] + k_{-28}[AA']$$

$$\frac{dBB'}{dt} = -k_{29}[BB'][T_2] + k_{-29}[T_2B][B'] + k_{35}[B][B'] - k_{-35}[BB']$$

$$\frac{dT_2}{dt} = -k_{29}[BB'][T_2] + k_{-29}[T_2B][B'] + k_{36}[H_{1b}]\,[T_2S] - k_{-36}[T_2][H_{1b}S]$$

$$\frac{dT_2B}{dt} = k_{29}[BB'][T_2] - k_{-29}[T_2B][B'] - k_{30}[T_2B][H_2] + k_{-30}[T_2BH_2] + k_{32}[T_2BH_{2a}H_{2b}] - k_{-32}[T_2B][H_{2a}H_{2b}]\,] - k_{34}[T_2B][H_{2a}] + k_{-34}[T_2H_{2a}][B]$$

$$\frac{dB'}{dt} = k_{29}[BB'][T_2] - k_{-29}[T_2B][B'] - k_{35}[B][B'] + k_{-35}[BB']$$

$$\frac{dH_2}{dt} = -k_{30}[T_2B][H_2] + k_{-30}[T_2BH_2]$$

$$\frac{dT_2BH_2}{dt} = k_{30}[T_2B][H_2] - k_{-30}[T_2BH_2] - k_{31}[T_2BH_2]$$

$$\frac{dT_2BH_{2aH2b}}{dt} = k_{31}[T_2BH_2] - k_{32}[T_2BH_{2a}H_{2b}] + k_{-32}[T_2B][H_{2a}H_{2b}]$$

$$\frac{dH_{2aH2b}}{dt} = k_{32}[T_2BH_{2a}H_{2b}] - k_{-32}[T_2B][H_{2a}H_{2b}] - k_{33}[H_{2a}H_{2b}] + k_{-33}[H_{2a}][H_{2b}]$$

$$\frac{dH_{2a}}{dt} = k_{33}[H_{2a}H_{2b}] - k_{-33}[H_{2a}][H_{2b}] - k_{34}[T_2B][H_{2a}] + k_{-34}[T_2H_{2a}][B]$$

$$\frac{dH_{2b}}{dt} = k_{33}[H_{2a}H_{2b}] - k_{-33}[H_{2a}][H_{2b}]$$

$$\frac{dT_2H_{2a}}{dt} = k_{34}[T_2B][H_{2a}] - k_{-34}[T_2H_{2a}][B]$$

$$\frac{dB}{dt} = k_{34}[T_2B][H_{2a}] - k_{-34}[T_2H_{2a}][B] - k_{35}[B][B'] + k_{-35}[BB']$$

$$\frac{dT_2S}{dt} = -k_{36}[H_{1b}]\,[T_2S] + k_{-36}[T_2][H_{1b}S]$$

$$\frac{dH_{1bS}}{dt} = k_{36}[H_{1b}]\,[T_2S] - k_{-36}[T_2][H_{1b}S]$$

Supplementary Figure 18. Computational simulation of the dissipative cascaded bilayer module I and module II shown in Figure 2. The kinetic scheme of the reactions associated with the time-dependent concentration changes during the

dissipative transitions of Mg^{2+}-ion-dependent DNAzymes T_1/A and T_2/B are summarized in above equations. Knowing the time-dependent concentration changes of the free unquenched fluorophores Cy5 and FAM, we computationally simulated the time-dependent concentration changes by using Matlab R2020a.

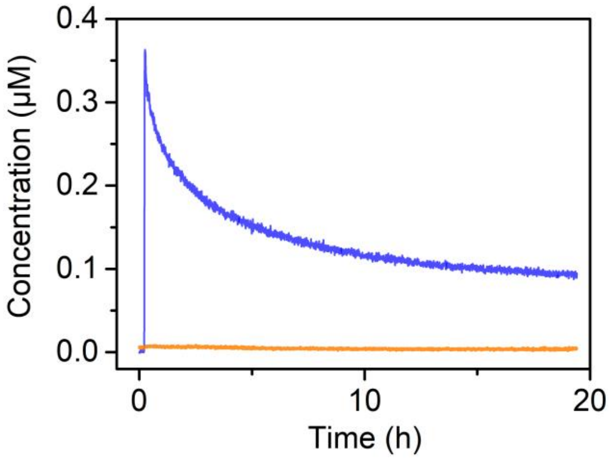

Supplementary Figure 19. Time-dependent concentration changes of cascaded system consisting of module I and II, where the coupler unit S/T_2 was excluded. The added trigger T_1 only initiates the module I (blue curve) and then leads to its dissipative behavior, no activation of the module II (orange curve), demonstrating the significance of the coupler element to intercommunicate the layers.

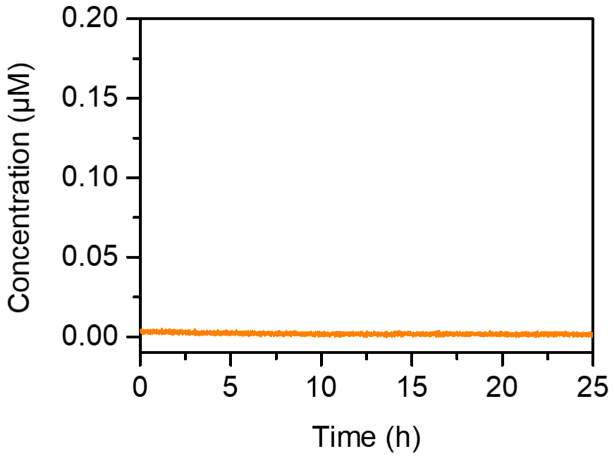

Supplementary Figure 20. Time-dependent dissipative concentration changes of separated module II in the presence of the coupler S/T$_2$, 1.2 μM. The result demonstrates that the S/T$_2$ had no effect on the separated module II, indicating that the bilayer cascaded dissipative behavior of the module II originates, indeed, from the cascaded intercommunication between the two modules.

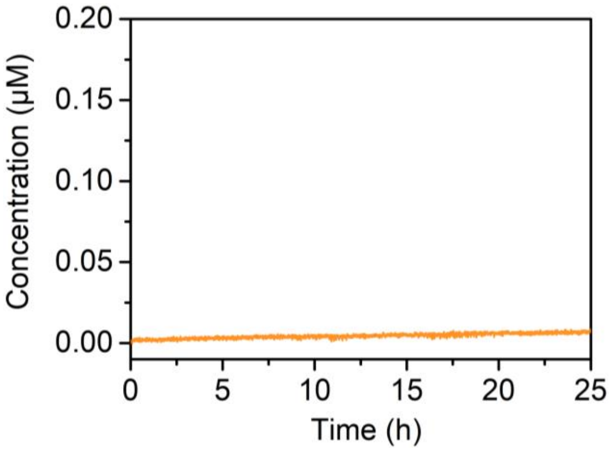

Supplementary Figure 21. Time-dependent dissipative concentration changes of separated module II in the presence of the fuel strand T_1, 1.2 μM. The result demonstrates that the trigger T_1 had no effect on the separated module II, indicating that the bilayer cascaded dissipative behavior of the module II originates, indeed, from the cascaded intercommunication between the two modules.

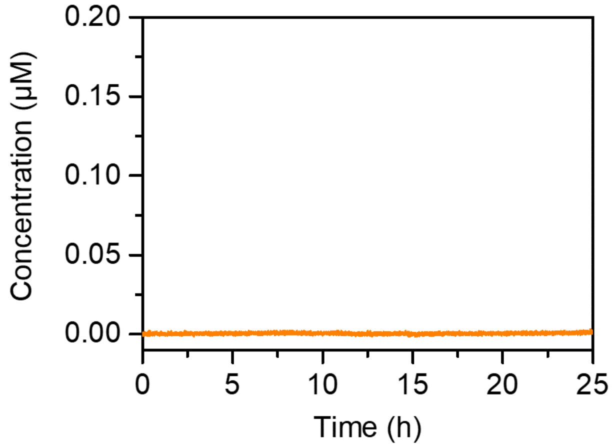

Supplementary Figure 22. Time-dependent dissipative concentration changes of separated module II in the presence of the H_1, 3 μM. The result demonstrates that the H_1 had no effect on the separated module II, indicating that the bilayer cascaded dissipative behavior of the module II originates, indeed, from the cascaded intercommunication between the two modules.

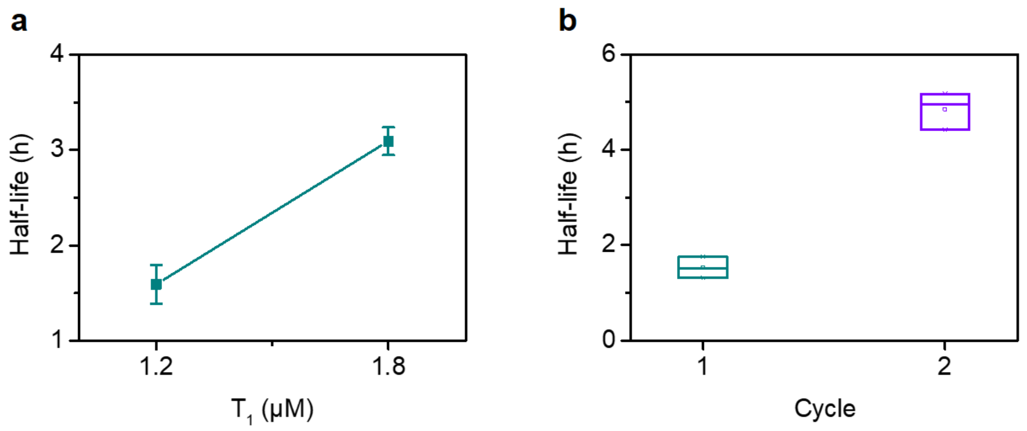

Supplementary Figure 23. Half-life of the DNAzyme T_1/A of the two-layer cascade with module I and module II. a, Half-life of the DNAzyme T_1/A of the two-layer cascade at variable concentrations of the trigger T_1. **b,** Half-life of the DNAzyme T_1/A of the two-layer cascade in the first and the second cycle showing in Figure 2**f**. Error bars were derived from N = 3 experiments.

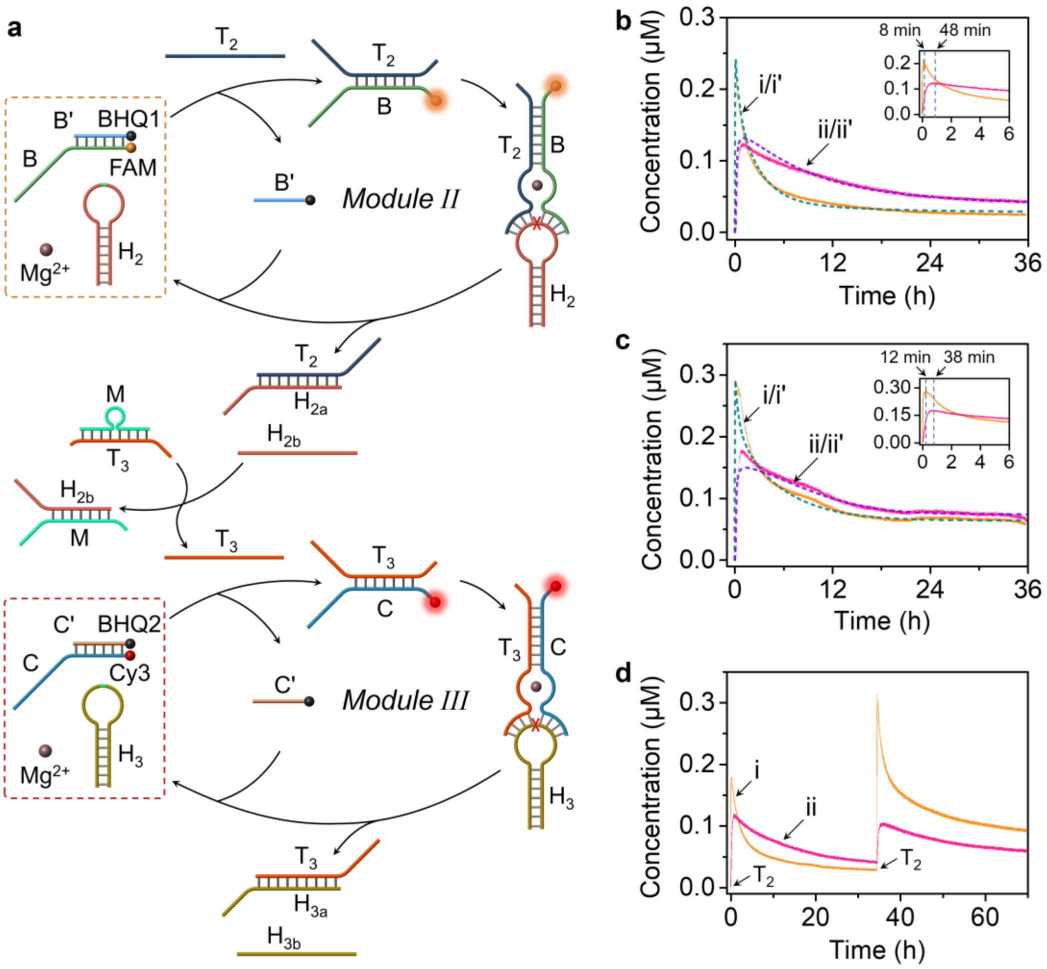

Supplementary Figure 24. Triggered operation of a two-layer transient DNAzyme cascade. **a**, Schematic T_2-triggered operation of a two-layer transient DNAzyme cascade where the T_2-triggered operation of the module II yields transient DNAzyme T_2/B that lead upon cleavage of hairpin H_2 to the activation of the M/T_3 coupler to yield the trigger T_3 activating the second layer module that yield the transient DNAzyme T_3/C. The dynamic cascaded two-layer network is followed by the dynamic fluorescence changes associated with the FAM and Cy3 fluorophores linked to respective DNAzyme. **b**, (i) Transient concentration changes of the DNAzyme T_2/B - solid line and (i') computationally simulated transient using the kinetic model presented in Supplementary Figure 27. (ii) Transient concentration changes of DNAzyme T_3/C - solid line and (ii') computationally simulated transient concentration changes. Results correspond to the operation of the bilayer cascade activated by T_2: 1.2 μM (BB' 0.6 μM, H_2 3 μM, M/T_3 1.2 μM, CC' 0.2 μM, H_3 1 μM). **c**, Transient predicted concentration changes of DNAzyme T_2/B (curve (i'), dashed line) and DNAzyme T_3/C (curve (ii'), dashed line) upon triggering the bilayer cascade using T_2 = 1.8 μM (BB' 0.6 μM, H_2 3 μM, M/T_3 1.8 μM, CC' 0.2 μM, H_3 1 μM). The solid lines (i) and (ii) correspond to the experimental transient concentration changes of DNAzyme T_2/B and DNAzyme T_3/C upon the T_2-triggered (1.8 μM) operation of the two-layer cascade, respectively. Insets in **b** and **c** correspond to the transient concentration changes the DNAzyme constituents at short time-scale of evolution and depletion of the intermediate DNAzyme. **d**, Cyclic

transient operation of the bilayer DNAzyme cascade: in terms of transient concentration changes of the dissipating DNAzymes. (Curve (i) - DNAzyme T_2/B; curve (ii) - DNAzyme T_3/C).

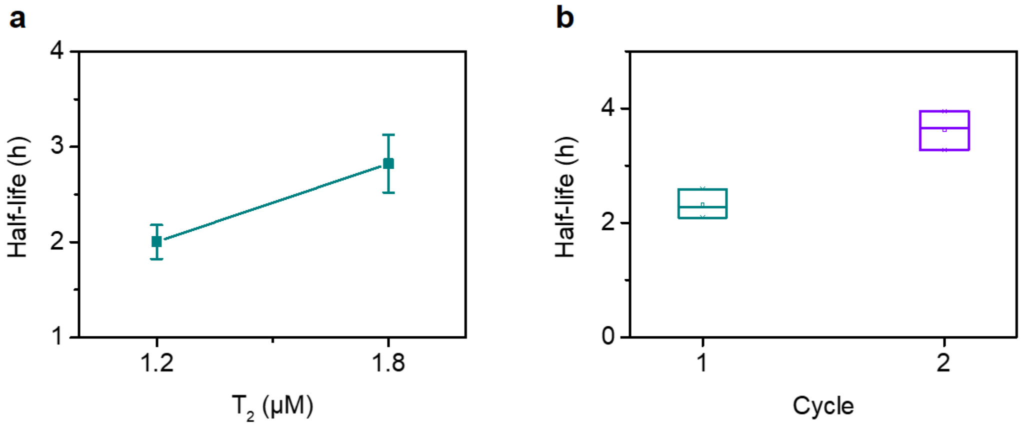

Supplementary Figure 25. Half-life of the DNAzyme T_2/B of the two-layer cascade with module II and module III. a, Half-life of the DNAzyme T_2/B of the two-layer cascade at variable concentrations of the trigger T_2. **b,** Half-life of the DNAzyme T_2/B of the two-layer cascade in the first and the second cycle showing in Supplementary Figure 24**d**. Error bars were derived from N = 3 experiments.

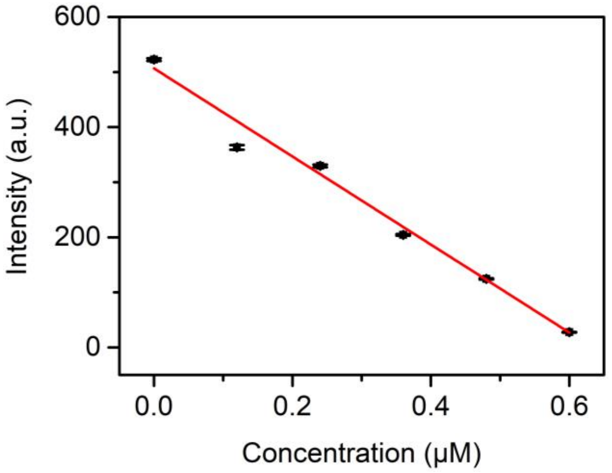

Supplementary Figure 26. Calibration curve corresponding to the fluorescence intensities of strand B (0.6 μM), in the presence of variable concentrations of the B'. The curve is fit linearly and the $r^2 = 0.99437$.

Kinetic equations of the dissipative cascaded bilayer module II and module III shown in Supplementary Figure 24:

$$BB' + T_2 \underset{k_{-37}}{\overset{k_{37}}{\rightleftharpoons}} T_2B + B' \quad (37)$$

$$T_2B + H_2 \underset{k_{-38}}{\overset{k_{38}}{\rightleftharpoons}} T_2BH_2 \quad (38)$$

$$T_2BH_2 \xrightarrow{k_{39}} T_2BH_{2a}H_{2b} \quad (39)$$

$$T_2BH_{2a}H_{2b} \underset{k_{-40}}{\overset{k_{40}}{\rightleftharpoons}} T_2B + H_{2a}H_{2b} \quad (40)$$

$$H_{2a}H_{2b} \underset{k_{-41}}{\overset{k_{41}}{\rightleftharpoons}} H_{2a} + H_{2b} \quad (41)$$

$$T_2B + H_{2a} \underset{k_{-42}}{\overset{k_{42}}{\rightleftharpoons}} T_2H_{2a} + B \quad (42)$$

$$B + B' \underset{k_{-43}}{\overset{k_{43}}{\rightleftharpoons}} BB' \quad (43)$$

$$H_{2b} + T_3M \underset{k_{-51}}{\overset{k_{51}}{\rightleftharpoons}} T_3 + H_{2b}M \quad (51)$$

$$CC' + T_3 \underset{k_{-44}}{\overset{k_{44}}{\rightleftharpoons}} T_3C + C' \quad (44)$$

$$T_3C + H_3 \underset{k_{-45}}{\overset{k_{45}}{\rightleftharpoons}} T_3CH_3 \quad (45)$$

$$T_3CH_3 \xrightarrow{k_{46}} T_3CH_{3a}H_{3b} \quad (46)$$

$$T_3CH_{3a}H_{3b} \underset{k_{-47}}{\overset{k_{47}}{\rightleftharpoons}} T_3C + H_{3a}H_{3b} \quad (47)$$

$$H_{3a}H_{3b} \underset{k_{-48}}{\overset{k_{48}}{\rightleftharpoons}} H_{3a} + H_{3b} \quad (48)$$

$$T_3C + H_{3a} \underset{k_{-49}}{\overset{k_{49}}{\rightleftharpoons}} T_3H_{3a} + C \quad (49)$$

$$C + C' \underset{k_{-50}}{\overset{k_{50}}{\rightleftharpoons}} CC' \quad (50)$$

Derivatives:

$$\frac{dBB'}{dt} = -k_{37}[BB'][T_2] + k_{-37}[T_2B][B'] + k_{43}[B][B'] - k_{-43}[BB']$$

$$\frac{dT_2}{dt} = -k_{37}[BB'][T_2] + k_{-37}[T_2B][B']$$

$$\frac{dT_2B}{dt} = k_{37}[BB'][T_2] - k_{-37}[T_2B][B'] - k_{38}[T_2B][H_2] + k_{-38}[T_2BH_2] + k_{40}[T_2BH_{2a}H_{2b}] - k_{-40}[T_2B][H_{2a}H_{2b}]\ ] - k_{42}[T_2B][H_{2a}] + k_{-42}[T_2H_{2a}][B]$$

$$\frac{dB'}{dt} = k_{37}[BB'][T_2] - k_{-37}[T_2B][B'] - k_{43}[B][B'] + k_{-43}[BB']$$

$$\frac{dH_2}{dt} = -k_{38}[T_2B][H_2] + k_{-38}[T_2BH_2]$$

$$\frac{dT_2BH_2}{dt} = k_{38}[T_2B][H_2] - k_{-38}[T_2BH_2] - k_{39}[T_2BH_2]$$

$$\frac{dT_2BH_{2aH2b}}{dt} = k_{39}[T_2BH_2] - k_{40}[T_2BH_{2a}H_{2b}] + k_{-40}[T_2B][H_{2a}H_{2b}]$$

$$\frac{dH_{2a}H_{2b}}{dt} = k_{40}[T_2BH_{2a}H_{2b}] - k_{-40}[T_2B][H_{2a}H_{2b}] - k_{41}[H_{2a}H_{2b}] + k_{-41}[H_{2a}][H_{2b}]$$

$$\frac{dH_{2a}}{dt} = k_{41}[H_{2a}H_{2b}] - k_{-41}[H_{2a}][H_{2b}] - k_{42}[T_2B][H_{2a}] + k_{-42}[T_2H_{2a}][B]$$

$$\frac{dH_{2b}}{dt} = k_{41}[H_{2a}H_{2b}] - k_{-41}[H_{2a}][H_{2b}] - k_{51}[H_{2b}]\ [T_3M] + k_{-51}[T_3][H_{2b}M]$$

$$\frac{dT_2H_{2a}}{dt} = k_{42}[T_2B][H_{2a}] - k_{-42}[T_2H_{2a}][B]$$

$$\frac{dB}{dt} = k_{42}[T_2B][H_{2a}] - k_{-42}[T_2H_{2a}][B] - k_{43}[B][B'] + k_{-43}[BB']$$

$$\frac{dCC'}{dt} = -k_{44}[CC'][T_3] + k_{-44}[T_3C][C'] + k_{50}[C][C'] - k_{-50}[CC']$$

$$\frac{dT_3}{dt} = -k_{44}[CC'][T_3] + k_{-44}[T_3C][C'] + k_{51}[H_{2b}]\ [T_3M] - k_{-51}[T_3][H_{2b}M]$$

$$\frac{dT_3C}{dt} = k_{44}[CC'][T_3] - k_{-44}[T_3C][C'] - k_{45}[T_3C][H_3] + k_{-45}[T_3CH_3] + k_{47}[T_3CH_{3a}H_{3b}] - k_{-47}[T_3C][H_{3a}H_{3b}]\ ] - k_{49}[T_3C][H_{3a}] + k_{-49}[T_3H_{3a}][C]$$

$$\frac{dC'}{dt} = k_{44}[CC'][T_3] - k_{-44}[T_3C][C'] - k_{50}[C][C'] + k_{-50}[CC']$$

$$\frac{dH_3}{dt} = -k_{45}[T_3C][H_3] + k_{-45}[T_3CH_3]$$

$$\frac{dT_3CH_3}{dt} = k_{45}[T_3C][H_3] - k_{-45}[T_3CH_3] - k_{46}[T_3CH_3]$$

$$\frac{dT_3CH_{3aH3b}}{dt} = k_{46}[T_3CH_3] - k_{47}[T_3CH_{3a}H_{3b}] + k_{-47}[T_3C][H_{3a}H_{3b}]$$

$$\frac{dH_{3a}H_{3b}}{dt} = k_{47}[T_3CH_{3a}H_{3b}] - k_{-47}[T_3\ C][H_{3a}H_{3b}] - k_{48}[H_{3a}H_{3b}] + k_{-48}[H_{3a}][H_{3b}]$$

$$\frac{dH_{3a}}{dt} = k_{48}[H_{3a}H_{3b}] - k_{-48}[H_{3a}][H_{3b}] - k_{49}[T_3C][H_{3a}] + k_{-49}[T_3H_{3a}][C]$$

$$\frac{dH_{3b}}{dt} = k_{48}[H_{3a}H_{3b}] - k_{-48}[H_{3a}][H_{3b}]$$

$$\frac{dT_3H_{3a}}{dt} = k_{49}[T_3C][H_{3a}] - k_{-49}[T_3H_{3a}][C]$$

$$\frac{dC}{dt} = k_{49}[T_3C][H_{3a}] - k_{-49}[T_3H_{3a}][C] - k_{50}[C][C'] + k_{-50}[CC']$$

$$\frac{dT_3M}{dt} = -k_{51}[H_{2b}]\ [T_3M] + k_{-51}[T_3][H_{2b}M]$$

$$\frac{dH_{2bM}}{dt} = k_{51}[H_{2b}]\ [T_3M] - k_{-51}[T_3][H_{2b}M]$$

Supplementary Figure 27. Computational simulation of the dissipative cascaded bilayer module II and module III shown in Supplementary Figure 24. The kinetic scheme of the reactions associated with the time-dependent concentration changes

during the dissipative transitions of Mg^{2+}-ion-dependent DNAzymes T_2/B and T_3/C are summarized in above equations. Knowing the time-dependent concentration changes of the free unquenched fluorophores FAM and Cy3, we computationally simulated the time-dependent concentration changes by using Matlab R2020a.

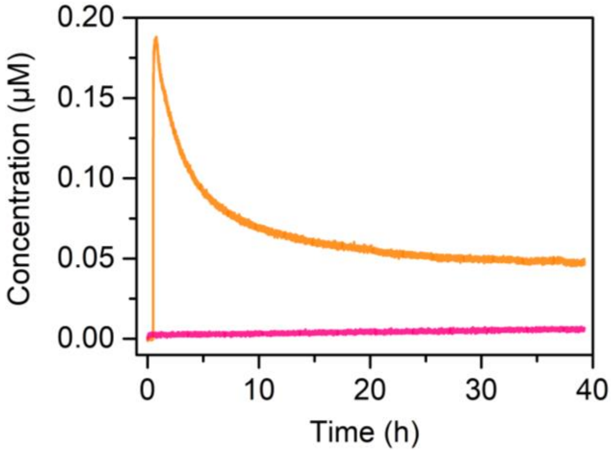

Supplementary Figure 28. Time-dependent concentration changes of cascaded system consisting of module II and III, where the coupler unit M/T_3 was excluded. The added trigger T_2 only initiates the module II (orange curve) and then leads to its dissipative behavior, no activation of the module III (pink curve), demonstrating the significance of the coupler element M/T_3 to intercommunicate the layers.

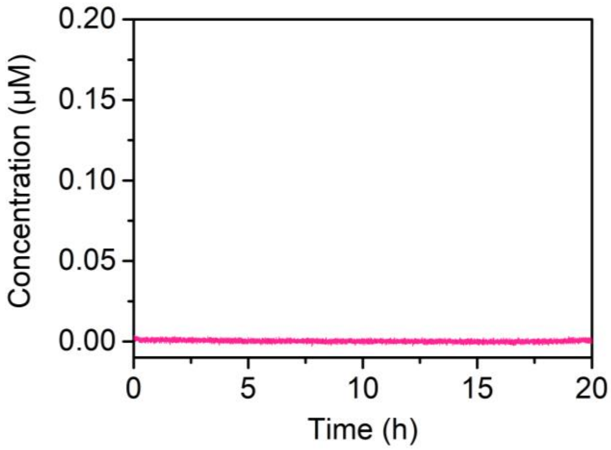

Supplementary Figure 29. Time-dependent dissipative concentration changes of separated module III in the presence of the fuel strand T_2, 1.2 μM. The result demonstrates that the trigger T_2 had no effect on the separated module III, indicating that the bilayer cascaded dissipative behavior of the module III originates, indeed, from the cascaded intercommunication between the two modules.

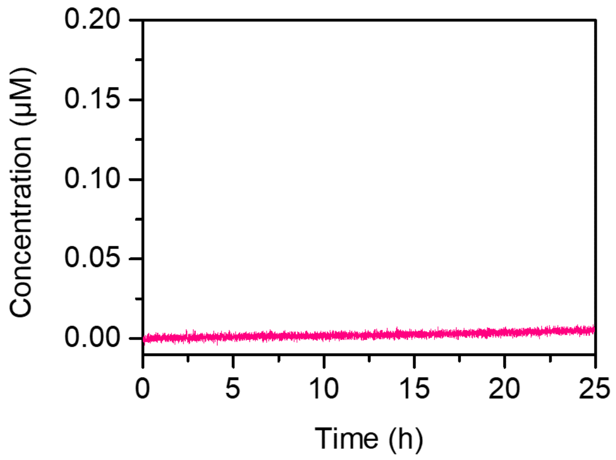

Supplementary Figure 30. Time-dependent dissipative concentration changes of separated module III in the presence of the M/T$_3$, 1.2 μM. The result demonstrates that the M/T$_3$ had no effect on the separated module III, indicating that the bilayer cascaded dissipative behavior of the module III originates, indeed, from the cascaded intercommunication between the two modules.

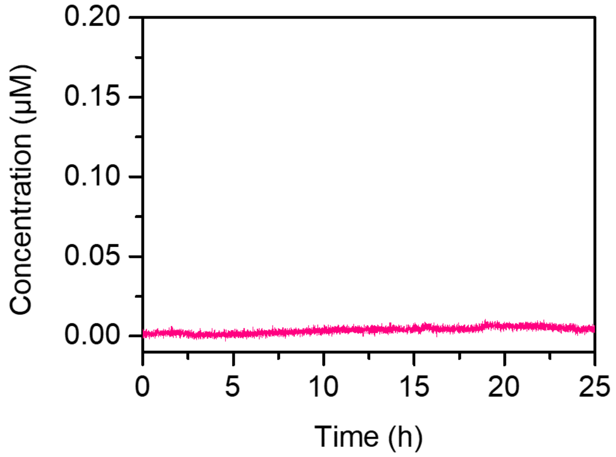

Supplementary Figure 31. Time-dependent dissipative concentration changes of separated module III in the presence of the H_2, 3 μM. The result demonstrates that the H_2 had no effect on the separated module III, indicating that the bilayer cascaded dissipative behavior of the module III originates, indeed, from the cascaded intercommunication between the two modules.

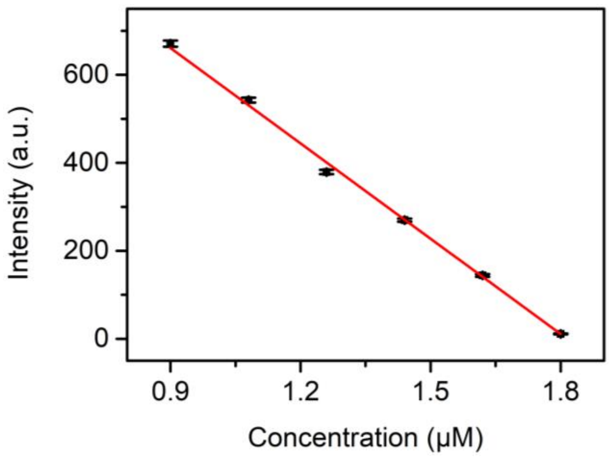

Supplementary Figure 32. Calibration curve corresponding to the fluorescence intensities of strand A (1.8 μM), in the presence of variable concentrations of the A'. The curve is fit linearly and the r^2 = 0.99875.

Kinetic equations of the dissipative cascaded three-layer module I, module II and module III shown in Figure 3:

$$AA' + T_1 \underset{k_{-52}}{\overset{k_{52}}{\rightleftharpoons}} T_1A + A' \quad (52)$$

$$T_1A + H_1 \underset{k_{-53}}{\overset{k_{53}}{\rightleftharpoons}} T_1AH_1 \quad (53)$$

$$T_1AH_1 \xrightarrow{k_{54}} T_1AH_{1a}H_{1b} \quad (54)$$

$$T_1AH_{1a}H_{1b} \underset{k_{-55}}{\overset{k_{55}}{\rightleftharpoons}} T_1A + H_{1a}H_{1b} \quad (55)$$

$$H_{1a}H_{1b} \underset{k_{-56}}{\overset{k_{56}}{\rightleftharpoons}} H_{1a} + H_{1b} \quad (56)$$

$$T_1A + H_{1a} \underset{k_{-57}}{\overset{k_{57}}{\rightleftharpoons}} T_1H_{1a} + A \quad (57)$$

$$A + A' \underset{k_{-58}}{\overset{k_{58}}{\rightleftharpoons}} AA' \quad (58)$$

$$H_{1b} + T_2S \underset{k_{-73}}{\overset{k_{73}}{\rightleftharpoons}} T_2 + H_{1b}S \quad (73)$$

$$BB' + T_2 \underset{k_{-59}}{\overset{k_{59}}{\rightleftharpoons}} T_2B + B' \quad (59)$$

$$T_2B + H_2 \underset{k_{-60}}{\overset{k_{60}}{\rightleftharpoons}} T_2BH_2 \quad (60)$$

$$T_2BH_2 \xrightarrow{k_{61}} T_2BH_{2a}H_{2b} \quad (61)$$

$$T_2BH_{2a}H_{2b} \underset{k_{-62}}{\overset{k_{62}}{\rightleftharpoons}} T_2B + H_{2a}H_{2b} \quad (62)$$

$$H_{2a}H_{2b} \underset{k_{-63}}{\overset{k_{63}}{\rightleftharpoons}} H_{2a} + H_{2b} \quad (63)$$

$$T_2B + H_{2a} \underset{k_{-64}}{\overset{k_{64}}{\rightleftharpoons}} T_2H_{2a} + B \quad (64)$$

$$B + B' \underset{k_{-65}}{\overset{k_{65}}{\rightleftharpoons}} BB' \quad (65)$$

$$H_{2b} + T_3M \underset{k_{-74}}{\overset{k_{74}}{\rightleftharpoons}} T_3 + H_{2b}M \quad (74)$$

$$CC' + T_3 \underset{k_{-66}}{\overset{k_{66}}{\rightleftharpoons}} T_3C + C' \quad (66)$$

$$T_3C + H_3 \underset{k_{-67}}{\overset{k_{67}}{\rightleftharpoons}} T_3CH_3 \quad (67)$$

$$T_3CH_3 \xrightarrow{k_{68}} T_3CH_{3a}H_{3b} \quad (68)$$

$$T_3CH_{3a}H_{3b} \underset{k_{-69}}{\overset{k_{69}}{\rightleftharpoons}} T_3C + H_{3a}H_{3b} \quad (69)$$

$$H_{3a}H_{3b} \underset{k_{-70}}{\overset{k_{70}}{\rightleftharpoons}} H_{3a} + H_{3b} \quad (70)$$

$$T_3C + H_{3a} \underset{k_{-71}}{\overset{k_{71}}{\rightleftharpoons}} T_3H_{3a} + C \quad (71)$$

$$C + C' \underset{k_{-72}}{\overset{k_{72}}{\rightleftharpoons}} CC' \quad (72)$$

$$H_{3b} + PW \underset{k_{-75}}{\overset{k_{75}}{\rightleftharpoons}} H_{3b}W + P \quad (75)$$

Derivatives:

$$\frac{dAA'}{dt} = -k_{52}[AA'][T_1] + k_{-52}[T_1A][A'] + k_{58}[A][A'] - k_{-58}[AA']$$

$$\frac{dT_1}{dt} = -k_{52}[AA'][T_1] + k_{-52}[T_1A][A']$$

$$\frac{dT_1A}{dt} = k_{52}[AA'][T_1] - k_{-52}[T_1A][A'] - k_{53}[T_1A][H_1] + k_{-53}[T_1AH_1] + k_{55}[T_1AH_{1a}H_{1b}] - k_{-55}[T_1A][H_{1a}H_{1b}]\ ] - k_{57}[T_1A][H_{1a}] + k_{-57}[T_1H_{1a}][A]$$

$$\frac{dA'}{dt} = k_{52}[AA'][T_1] - k_{-52}[T_1A][A'] - k_{58}[A][A'] + k_{-58}[AA']$$

$$\frac{dH_1}{dt} = -k_{53}[T_1A][H_1] + k_{-53}[T_1AH_1]$$

$$\frac{dT_1AH_1}{dt} = k_{53}[T_1A][H_1] - k_{-53}[T_1AH_1] - k_{54}[T_1AH_1]$$

$$\frac{dT_1AH_{1aH1b}}{dt} = k_{54}[T_1AH_1] - k_{55}[T_1AH_{1a}H_{1b}] + k_{-55}[T_1A][H_{1a}H_{1b}]$$

$$\frac{dH_{1aH1b}}{dt} = k_{55}[T_1AH_{1a}H_{1b}] - k_{-55}[T_1\ A][H_{1a}H_{1b}] - k_{56}[H_{1a}H_{1b}] + k_{-56}[H_{1a}][H_{1b}]$$

$$\frac{dH_{1a}}{dt} = k_{56}[H_{1a}H_{1b}] - k_{-56}[H_{1a}][H_{1b}] - k_{57}[T_1A][H_{1a}] + k_{-57}[T_1H_{1a}][A]$$

$$\frac{dH_{1b}}{dt} = k_{56}[H_{1a}H_{1b}] - k_{-56}[H_{1a}][H_{1b}] - k_{73}[H_{1b}]\ [T_2S] + k_{-73}[T_2][H_{1b}S]$$

$$\frac{dT_1H_{1a}}{dt} = k_{57}[T_1A][H_{1a}] - k_{-57}[T_1H_{1a}][A]$$

$$\frac{dA}{dt} = k_{57}[T_1A][H_{1a}] - k_{-57}[T_1H_{1a}][A] - k_{58}[A][A'] + k_{-58}[AA']$$

$$\frac{dBB'}{dt} = -k_{59}[BB'][T_2] + k_{-59}[T_2B][B'] + k_{65}[B][B'] - k_{-65}[BB']$$

$$\frac{dT_2}{dt} = -k_{59}[BB'][T_2] + k_{-59}[T_2B][B'] + k_{73}[H_{1b}]\ [T_2S] - k_{-73}[T_2][H_{1b}S]$$

$$\frac{dT_2B}{dt} = k_{59}[BB'][T_2] - k_{-59}[T_2B][B'] - k_{60}[T_2B][H_2] + k_{-60}[T_2BH_2] + k_{62}[T_2BH_{2a}H_{2b}] - k_{-62}[T_2B][H_{2a}H_{2b}]\ ] - k_{64}[T_2B][H_{2a}] + k_{-64}[T_2H_{2a}][B]$$

$$\frac{dB'}{dt} = k_{59}[BB'][T_2] - k_{-59}[T_2B][B'] - k_{65}[B][B'] + k_{-65}[BB']$$

$$\frac{dH_2}{dt} = -k_{60}[T_2B][H_2] + k_{-60}[T_2BH_2]$$

$$\frac{dT_2BH_2}{dt} = k_{60}[T_2B][H_2] - k_{-60}[T_2BH_2] - k_{61}[T_2BH_2]$$

$$\frac{dT_2BH_{2aH2b}}{dt} = k_{61}[T_2BH_2] - k_{62}[T_2BH_{2a}H_{2b}] + k_{-62}[T_2B][H_{2a}H_{2b}]$$

$$\frac{dH_{2a}H_{2b}}{dt} = k_{62}[T_2BH_{2a}H_{2b}] - k_{-62}[T_2B][H_{2a}H_{2b}] - k_{63}[H_{2a}H_{2b}] + k_{-63}[H_{2a}][H_{2b}]$$

$$\frac{dH_{2a}}{dt} = k_{63}[H_{2a}H_{2b}] - k_{-63}[H_{2a}][H_{2b}] - k_{64}[T_2B][H_{2a}] + k_{-64}[T_2H_{2a}][B]$$

$$\frac{dH_{2b}}{dt} = k_{63}[H_{2a}H_{2b}] - k_{-63}[H_{2a}][H_{2b}] - k_{74}[H_{2b}]\ [T_3M] + k_{-74}[T_3][H_{2b}M]$$

$$\frac{dT_2H_{2a}}{dt} = k_{64}[T_2B][H_{2a}] - k_{-64}[T_2H_{2a}][B]$$

$$\frac{dB}{dt} = k_{64}[T_2B][H_{2a}] - k_{-64}[T_2H_{2a}][B] - k_{65}[B][B'] + k_{-65}[BB']$$

$$\frac{dT_2S}{dt} = -k_{73}[H_{1b}]\ [T_2S] + k_{-73}[T_2][H_{1b}S]$$

$$\frac{dH_{1bS}}{dt} = k_{73}[H_{1b}]\ [T_2S] - k_{-73}[T_2][H_{1b}S]$$

$$\frac{dCC'}{dt} = -k_{66}[CC'][T_3] + k_{-66}[T_3C][C'] + k_{72}[C][C'] - k_{-72}[CC']$$

$$\frac{dT_3}{dt} = -k_{66}[CC'][T_3] + k_{-66}[T_3C][C'] + k_{74}[H_{2b}][T_3M] - k_{-74}[T_3][H_{2b}M]$$

$$\frac{dT_3C}{dt} = k_{66}[CC'][T_3] - k_{-66}[T_3C][C'] - k_{67}[T_3C][H_3] + k_{-67}[T_3CH_3] + k_{69}[T_3CH_{3a}H_{3b}] - k_{-69}[T_3C][H_{3a}H_{3b}]\,] - k_{71}[T_3C][H_{3a}] + k_{-71}[T_3H_{3a}][C]$$

$$\frac{dC'}{dt} = k_{66}[CC'][T_3] - k_{-66}[T_3C][C'] - k_{72}[C][C'] + k_{-72}[CC']$$

$$\frac{dH_3}{dt} = -k_{67}[T_3C][H_3] + k_{-67}[T_3CH_3]$$

$$\frac{dT_3CH_3}{dt} = k_{67}[T_3C][H_3] - k_{-67}[T_3CH_3] - k_{68}[T_3CH_3]$$

$$\frac{dT_3CH_{3aH3b}}{dt} = k_{68}[T_3CH_3] - k_{69}[T_3CH_{3a}H_{3b}] + k_{-69}[T_3C][H_{3a}H_{3b}]$$

$$\frac{dH_{3a}H_{3b}}{dt} = k_{69}[T_3CH_{3a}H_{3b}] - k_{-69}[T_3\,C][H_{3a}H_{3b}] - k_{70}[H_{3a}H_{3b}] + k_{-70}[H_{3a}][H_{3b}]$$

$$\frac{dH_{3a}}{dt} = k_{70}[H_{3a}H_{3b}] - k_{-70}[H_{3a}][H_{3b}] - k_{71}[T_3C][H_{3a}] + k_{-71}[T_3H_{3a}][C]$$

$$\frac{dH_{3b}}{dt} = k_{70}[H_{3a}H_{3b}] - k_{-70}[H_{3a}][H_{3b}]$$

$$\frac{dT_3H_{3a}}{dt} = k_{71}[T_3C][H_{3a}] - k_{-71}[T_3H_{3a}][C]$$

$$\frac{dC}{dt} = k_{71}[T_3C][H_{3a}] - k_{-71}[T_3H_{3a}][C] - k_{72}[C][C'] + k_{-72}[CC']$$

$$\frac{dT_3M}{dt} = -k_{74}[H_{2b}]\,[T_3M] + k_{-74}[T_3][H_{2b}M]$$

$$\frac{dH_{2bM}}{dt} = k_{74}[H_{2b}]\,[T_3M] - k_{-74}[T_3][H_{2b}M]$$

$$\frac{dPW}{dt} = -k_{75}[H_{3b}]\,[PW] + k_{-75}[P][H_{3b}W]$$

$$\frac{dH_{3bW}}{dt} = k_{75}[H_{3b}]\,[PW] - k_{-75}[P][H_{3b}W]$$

$$\frac{dP}{dt} = k_{75}[H_{3b}]\,[PW] - k_{-75}[P][H_{3b}W]$$

Supplementary Figure 33. Computational simulation of the dissipative cascaded three-layer module I, module II and module III shown in Figure 3. The kinetic scheme of the reactions associated with the time-dependent concentration changes during the dissipative transitions of Mg^{2+}-ion-dependent DNAzymes T_1/A, T_2/B and T_3/C are summarized in above equations. Knowing the time-dependent concentration changes of the free unquenched fluorophores Cy5, FAM and Cy3, we computationally simulated the time-dependent concentration changes by using Matlab R2020a.

Comparison of times for reaching the peak transient concentrations of DNAzyme T_2/B in the two-layer cascade and three-layer cascades

Figure 2d shows the peak transient concentration of T_2/B in the two-layer cascade is reached after 108 minutes. In turn, Figure 3i, shows that the peak transient concentration of the DNAzyme T_2/B is reached, in the three-layer cascade after a substantially shorter time-scale corresponding to 35 minutes. This apparent discrepancy is attributed to the fact that in the three-layer cascade the product of module II is consumed by displacing the unit M/T_3. As a result, the competitive binding of H_{2b} to H_{2a} is hindered and the recovery of the module II will be enhanced. Consequently, the peak concentration of module II will appear at a time (35 minutes) that is shorter than the time (108 minutes) observed in the absence of module III.

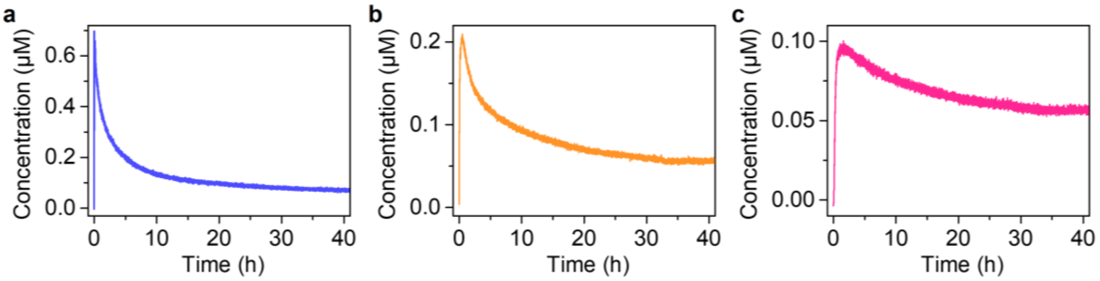

Supplementary Figure 34. Triggered operation of a three-layer, transient, DNAzyme cascade integrated by module I, II and III, in the absence of duplex P/W, where T_1-yields the transient formation of DNAzyme T_1/A (a) that activates the transient formation of DNAzyme T_2/B (b) that further triggers the transient operation of DNAzyme T_3/C (c), while the transient operations of DNAzyme T_1/A and T_2/B are unaffected, the transient recovery of the DNAzyme T_3/C is substantially slower. This may be attributed to the recombination of the fragmented products H_{3a} and H_{3b} with the constituent T_3/C, that inhibits the separation of T_3 that provides the driving force to recover the third layer. That is, the coupler W/P removes H_{3b} from the system, thus shifting the contents of the constituents to favor the displacement of T_3, and the fast recovery of the third layer.

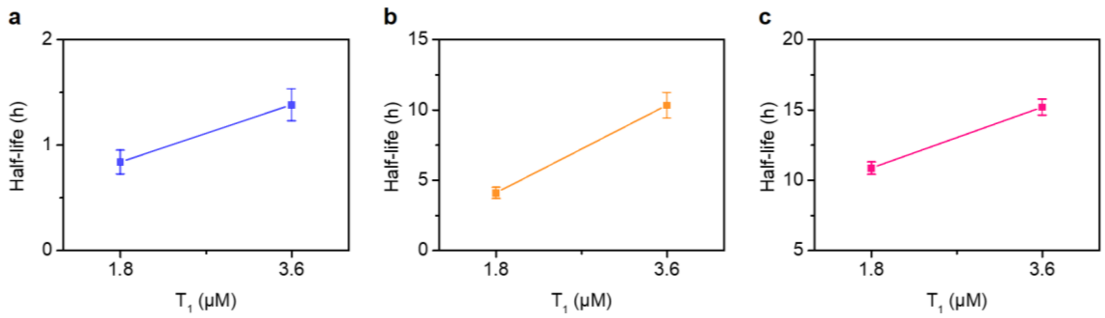

Supplementary Figure 35. Half-life of the DNAzymes T_1/A, T_2/B and T_3/C of the three-layer cascade with module I, module II and module III. a-c, Half-life of the DNAzymes T_1/A (**a**), T_2/B (**b**) and T_3/C (**c**) of the three-layer cascade at variable concentrations of the trigger T_1. Error bars were derived from N = 3 experiments.

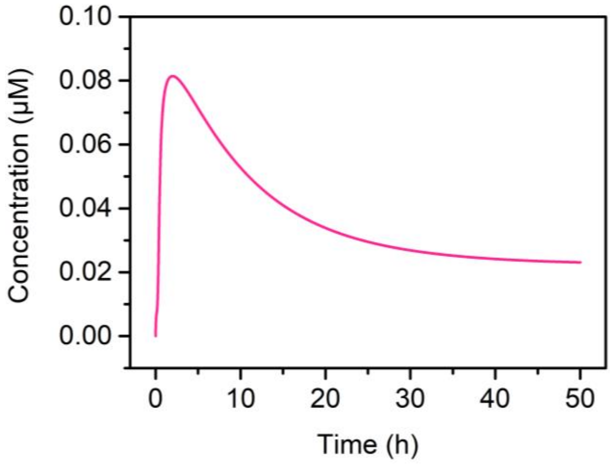

Supplementary Figure 36. The transient temporal formation and depletion of the constituent C' predicted by the kinetic model corresponding to the three-layered cascade formulated on page 40-42 and the resulting simulated rate constants provided in Supplementary Table 6.

Evaluation of the concentrations of the DNAzyme-coded strands G_1, G_2 and G_3

The experimental procedure to evaluate the concentrations of the coded strands followed by the following steps:

(i) We take samples out of the three-layer cascade at different time-intervals of operation.

(ii) We apply the replication/nicking machinery synthesizing the coded genes G_1, G_2, G_3 on each of the samples for a time-interval of six hours.

(iii) After completion of the synthesis of the coded genes, the substrates S_1, S_2, S_3 are added at an excess (2 μM), and the concentrations of the synthesized coded genes are quantitatively evaluated by following the rates of cleavage of the substrates for a short time interval (100 minutes). The derived concentrations represent the temporal behavior of the formation of the respective coded genes.

The experimental details are as follows:

Characterization of the coded strands, "model genes" replication guided by the transient cascade. Samples consisting of 30 μL solution of the operating three-layer cascaded system (trigger T_1, 3.6 μM were added) at variable intervals of the operating cascade were added into a mixture 100 μL that included the three DNA templates (0.01 μM each), 2 μL Klenow Fragment (3'→5' exo-), 2 μL Nt.BbvCI and dNTPs (0.3 mM) in 1× NEBuffer™ 2 (50 mM NaCl, 10 mM Tris-HCl, 10 mM $MgCl_2$, 1 mM DTT, pH 7.9) to react at 28 °C for 6 h. Subsequently, the solution was treated with the substrates S_1, S_2 and S_3 (2 μM each) and the time-dependent fluorescence intensity changes of the respective fluorophore labeled fragmented substrates were followed (Cy5, λ_{ex} = 635 nm, λ_{em} = 665 nm; FAM, λ_{ex} = 495 nm, λ_{em} = 518 nm; ROX, λ_{ex} = 588 nm, λ_{em} = 608 nm).

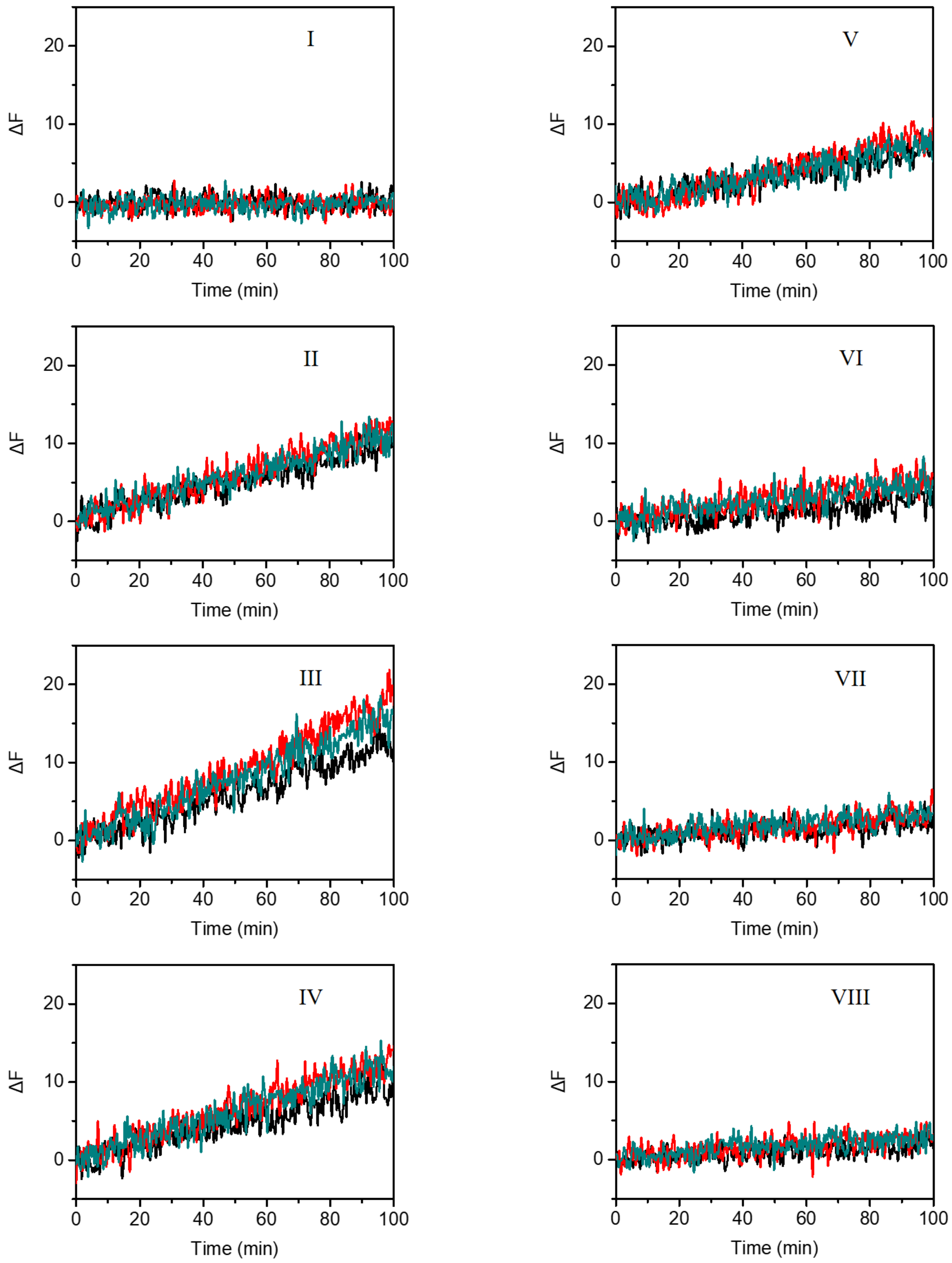

Supplementary Figure 37. Time-dependent catalytic activities of the DNAzyme G_1 generated by the functional coded gene-replication model in the presence of 3.6 μM T_1 at different time intervals: I, 0 h; II, 0.5 h; III, 1.33 h; IV, 6 h; V, 14 h; VI, 26 h; VII, 38 h; VIII, 50 h. Each measurement was repeated three times (N = 3).

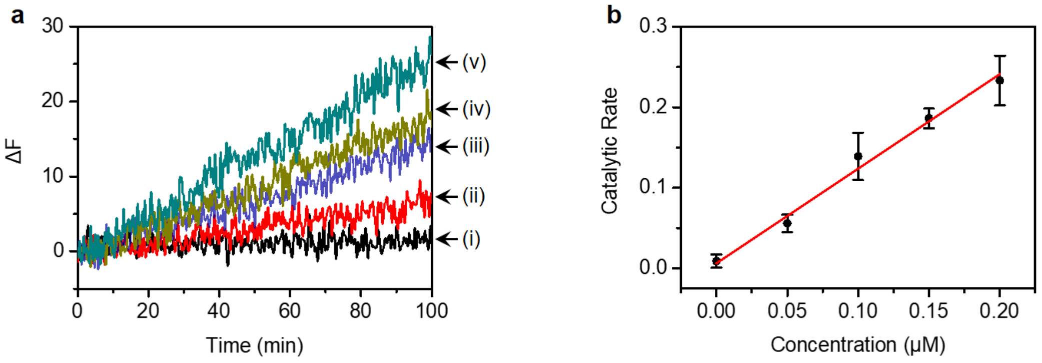

Supplementary Figure 38. Calibration Curve of the catalytic activities of DNAzyme G_1 to cleave substrate S_1 labeled with fluorophore Cy5 and quencher BHQ2. a, Time-dependent fluorescence changes generated by the cleavage of substrate S_1 with different concentrations of DNAzyme G_1. **b,** Calibration curve corresponding to the catalytic rates of the DNAzyme G_1 as a function of its concentrations (r^2 = 0.99079). Error bars were derived from N = 3 experiments.

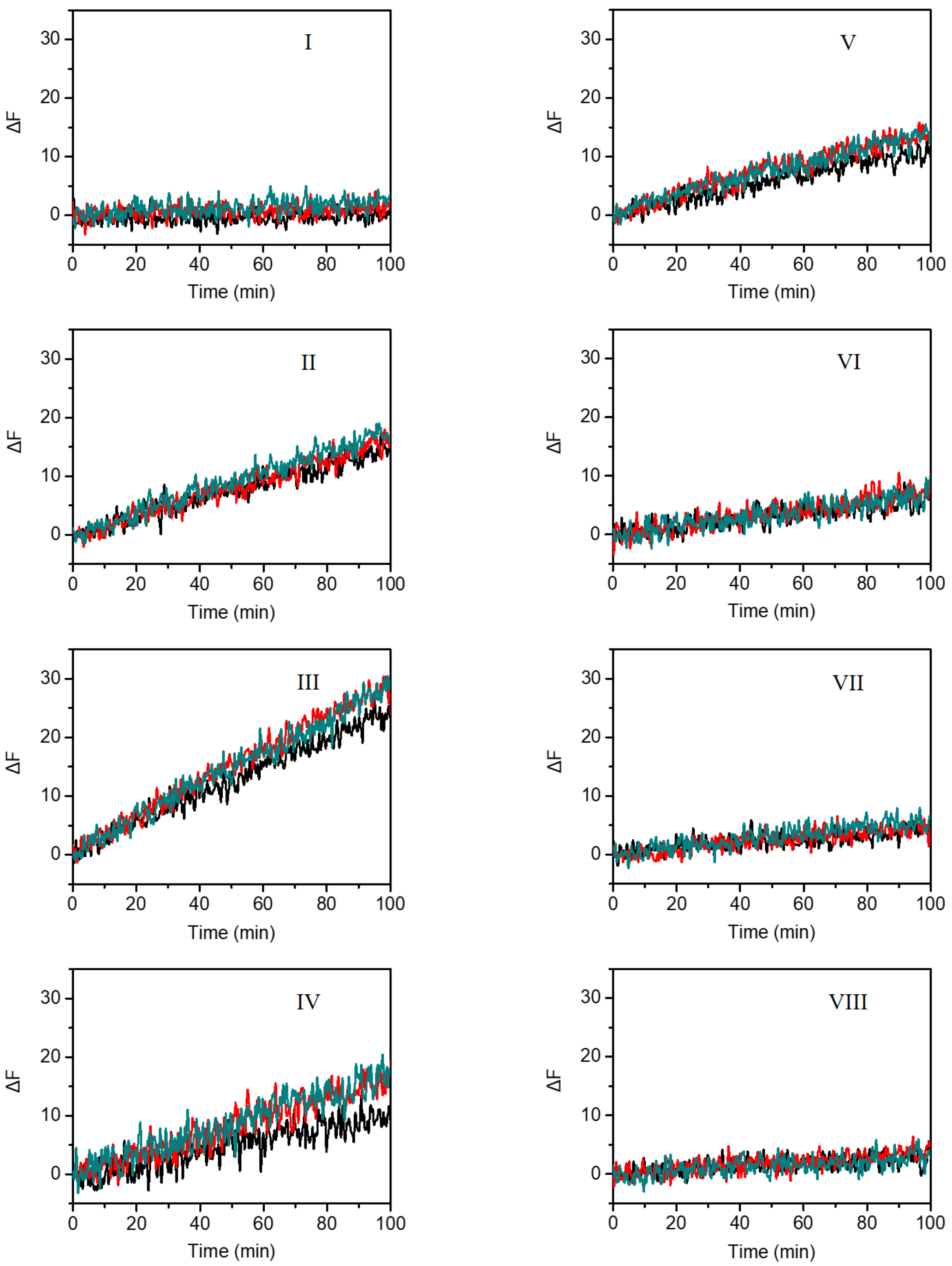

Supplementary Figure 39. Time-dependent catalytic activities of the DNAzyme G_2 generated by the functional coded gene-replication model in the presence of 3.6 μM T_1 at different time intervals: I, 0 h; II, 0.5 h; III, 1.33 h; IV, 6 h; V, 14 h; VI, 26 h; VII, 38 h; VIII, 50 h. Each measurement was repeated three times (N = 3).

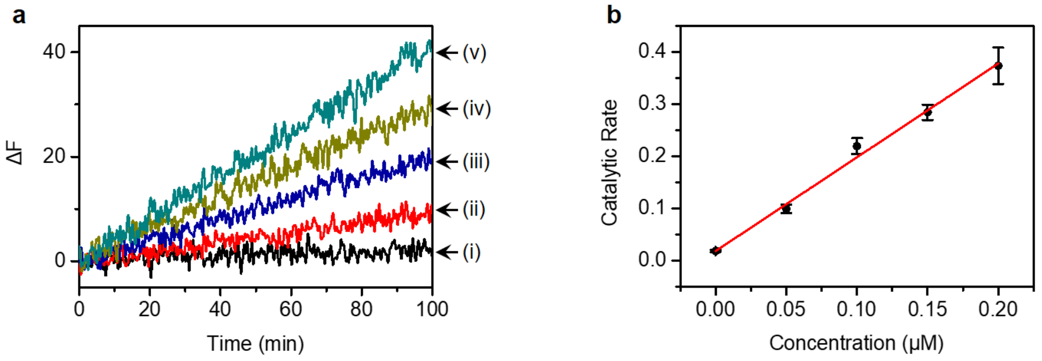

Supplementary Figure 40. Calibration Curve of the catalytic activities of DNAzyme G_2 to cleave substrate S_2 labeled with fluorophore ROX and quencher BHQ2. a, Time-dependent fluorescence changes generated by the cleavage of substrate S_2 with different concentrations of DNAzyme G_2. **b,** Calibration curve corresponding to the catalytic rates of the DNAzyme G_2 as a function of its concentrations (r^2 = 0.99354). Error bars were derived from N = 3 experiments.

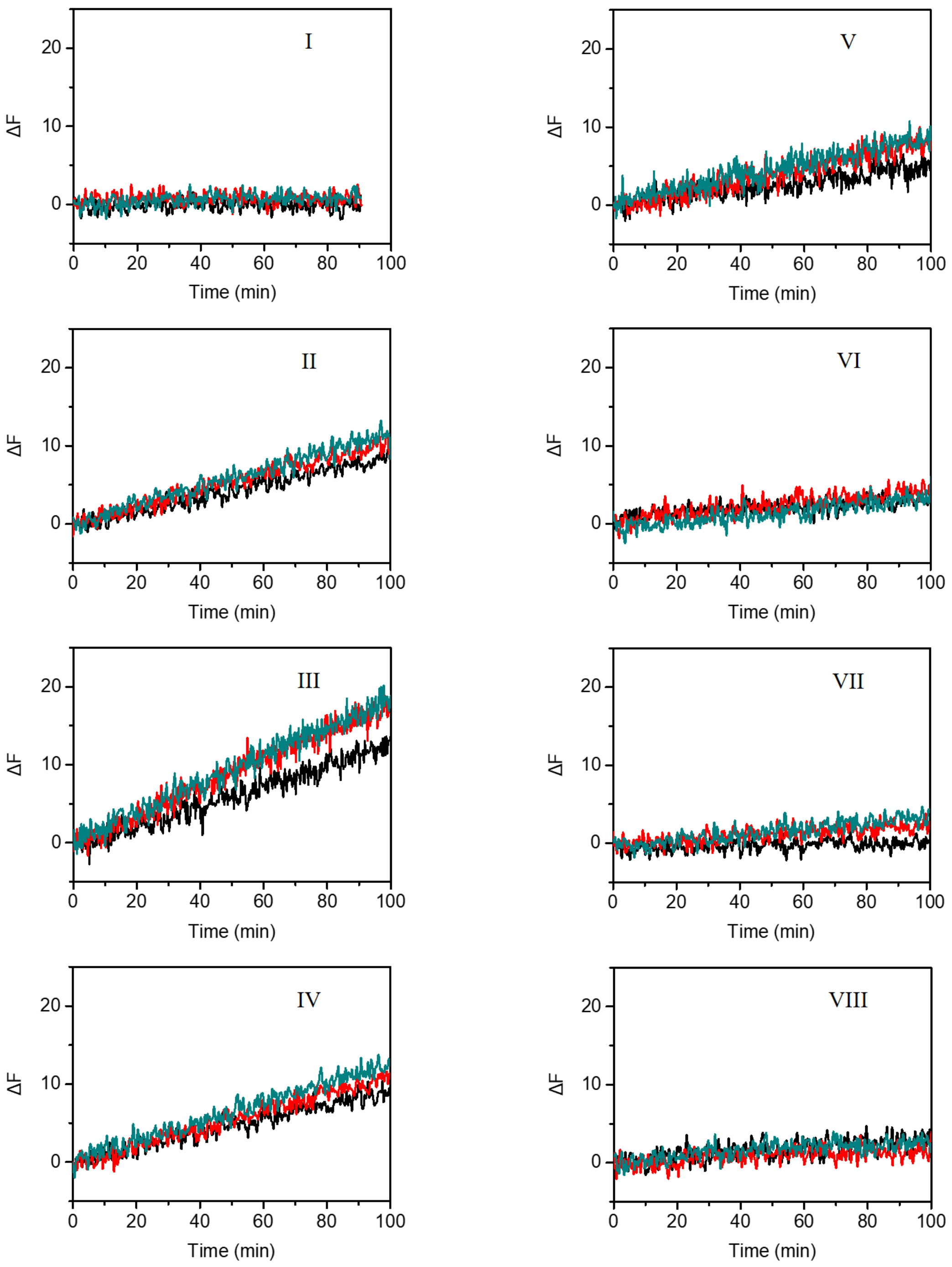

Supplementary Figure 41. Time-dependent catalytic activities of the DNAzyme G_3 generated by the functional coded gene-replication model in the presence of 3.6 μM T_1 at different time intervals: I, 0 h; II, 0.5 h; III, 1.33 h; IV, 6 h; V, 14 h; VI, 26 h; VII, 38 h; VIII, 50 h. Each measurement was repeated three times (N = 3).

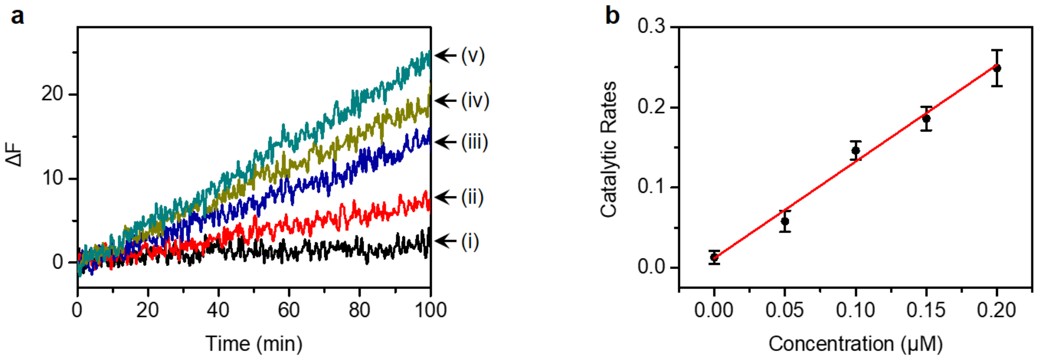

Supplementary Figure 42. Calibration Curve of the catalytic activities of DNAzyme G_3 to cleave substrate S_3 labeled with fluorophore FAM and quencher BHQ1. a, Time-dependent fluorescence changes generated by the cleavage of substrate S_3 with different concentrations of DNAzyme G_3. **b,** Calibration curve corresponding to the catalytic rates of the DNAzyme G_3 as a function of its concentrations (r^2 = 0.98217). Error bars were derived from N = 3 experiments.

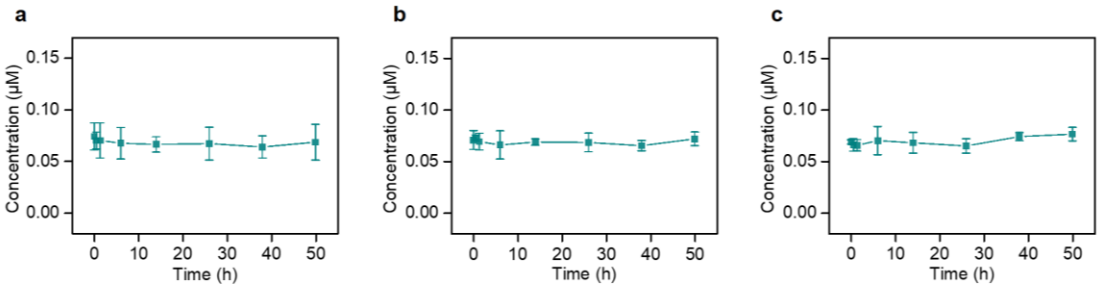

Supplementary Figure 43. Transient concentration changes of the DNAzyme model genes generated by the functional coded gene-replication model in the presence of constant concentrations of the strand C' (0.05 µM): a, DNAzyme G_1; b, DNAzyme G_2; c, DNAzyme G_3. Error bars were derived from N = 3 experiments.

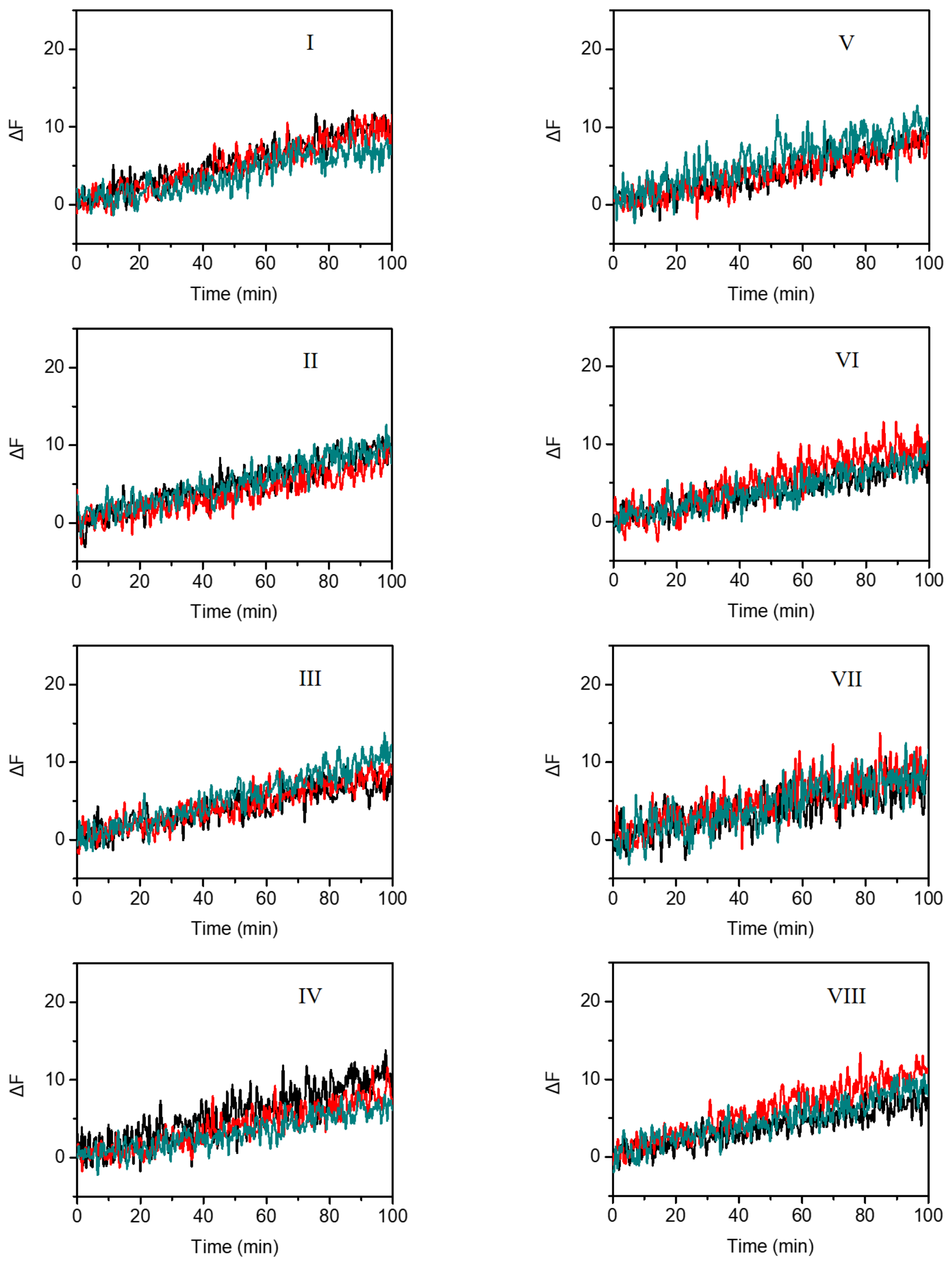

Supplementary Figure 44. Time-dependent catalytic activities of the DNAzyme G_1 generated by the functional coded gene-replication model in the presence of constant concentration of the strand C' (0.05 µM) at different time intervals: I, 0 h; II, 0.5 h; III, 1.33 h; IV, 6 h; V, 14 h; VI, 26 h; VII, 38 h; VIII, 50 h. Each measurement was repeated three times (N = 3).

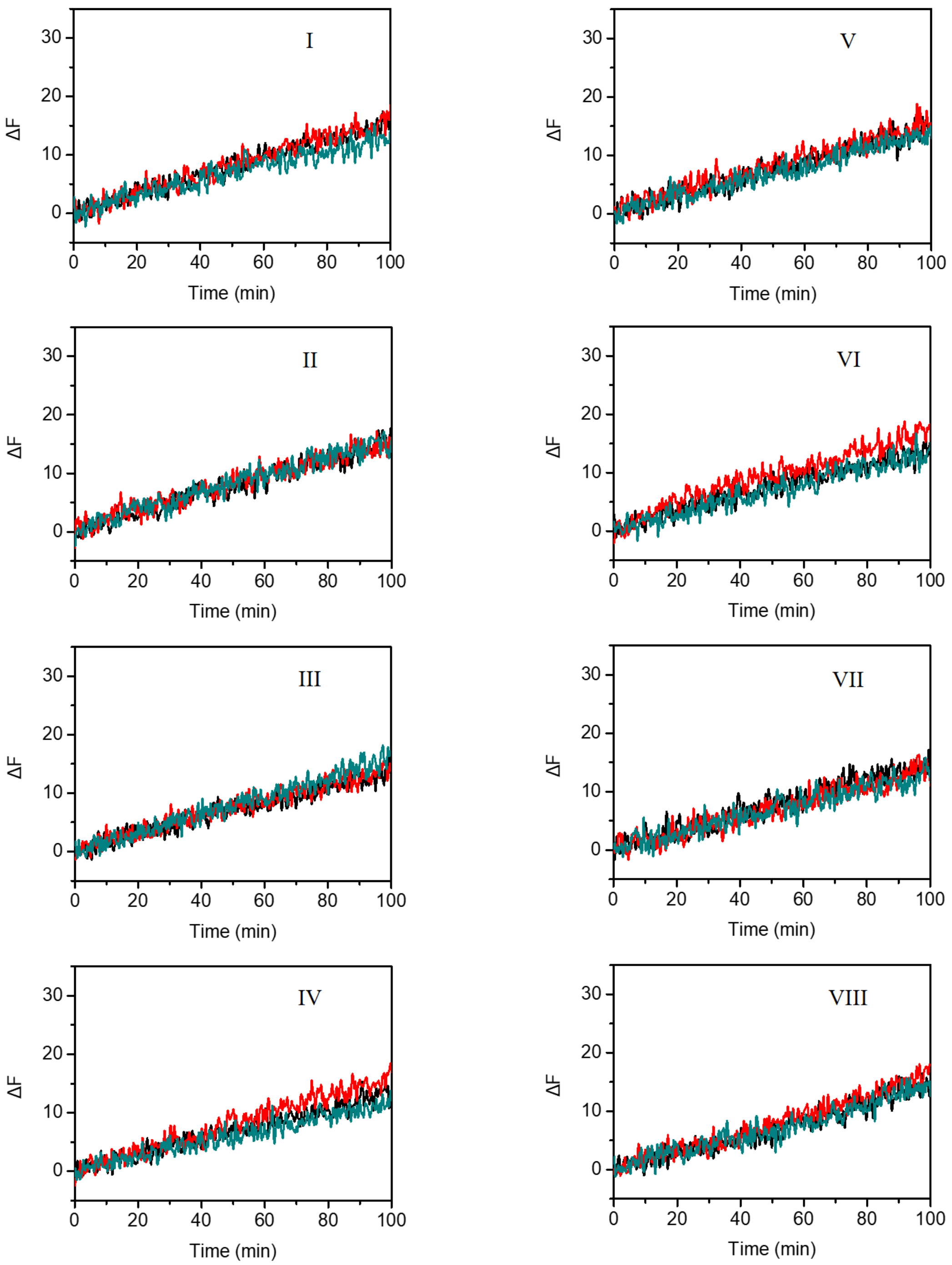

Supplementary Figure 45. Time-dependent catalytic activities of the DNAzyme G_2 generated by the functional coded gene-replication model in the presence of constant concentration of the strand C' (0.05 µM) at different time intervals: I, 0 h; II, 0.5 h; III, 1.33 h; IV, 6 h; V, 14 h; VI, 26 h; VII, 38 h; VIII, 50 h. Each measurement was repeated three times (N = 3).

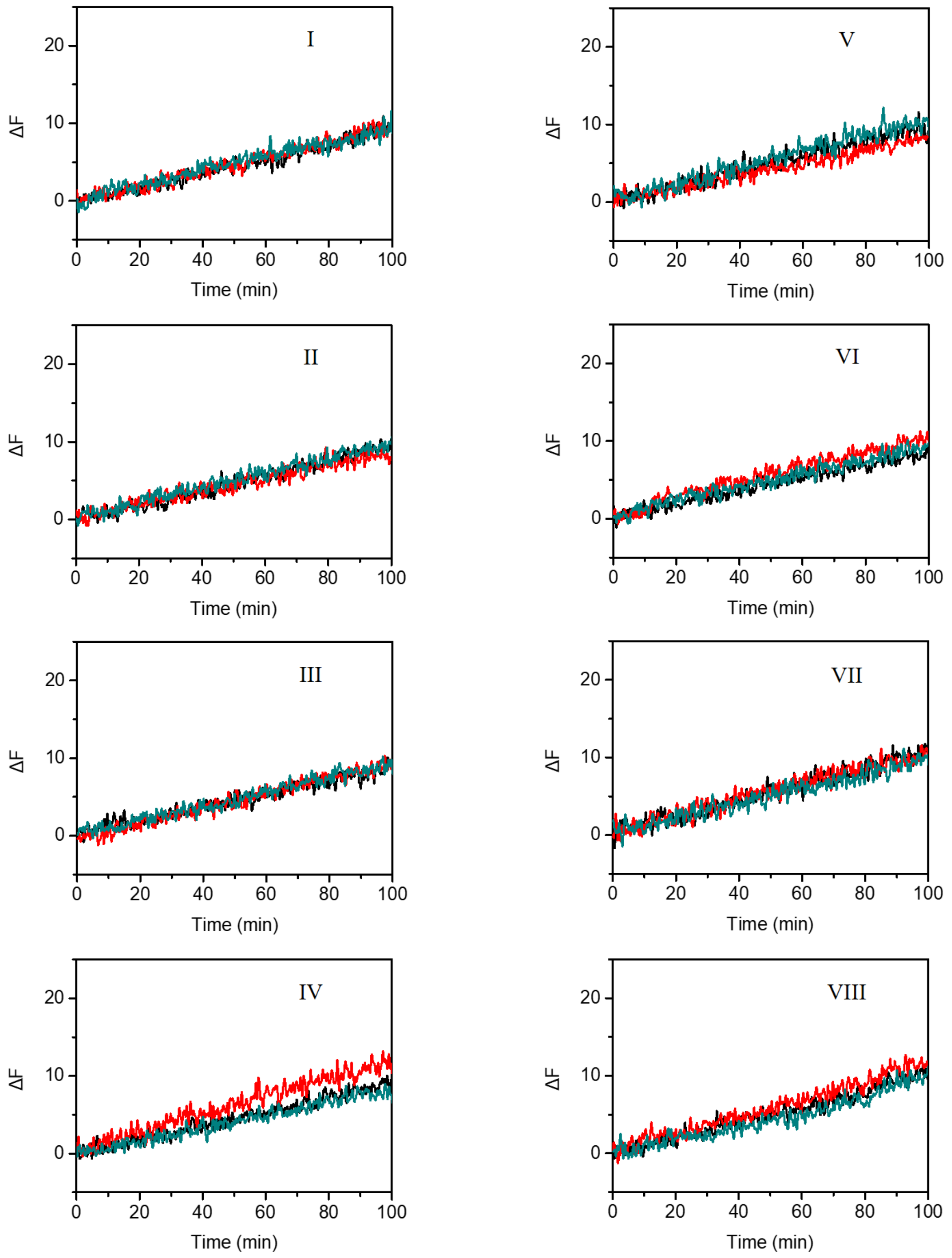

Supplementary Figure 46. Time-dependent catalytic activities of the DNAzyme G_3 generated by the functional coded gene-replication model in the presence of constant concentration of the strand C' (0.05 µM) at different time intervals: I, 0 h; II, 0.5 h; III, 1.33 h; IV, 6 h; V, 14 h; VI, 26 h; VII, 38 h; VIII, 50 h. Each measurement was repeated three times (N = 3).

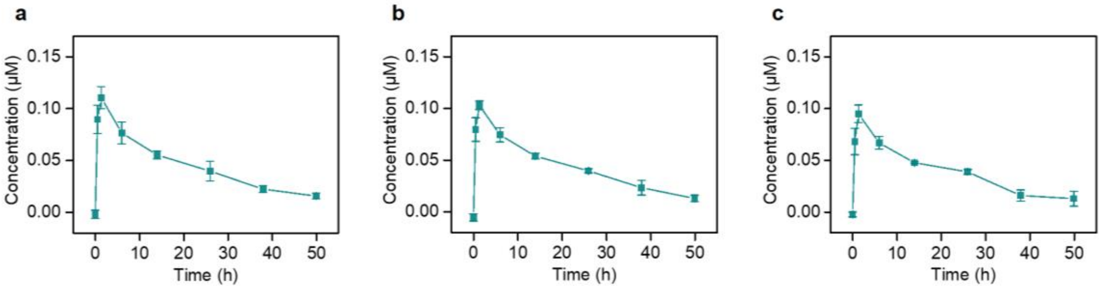

Supplementary Figure 47. Transient concentration changes of the DNAzyme model genes generated by the functional coded gene-replication model in the presence of 1.8 μM T_1 trigger: a, DNAzyme G_1; b, DNAzyme G_2; c, DNAzyme G_3. Error bars were derived from N = 3 experiments.

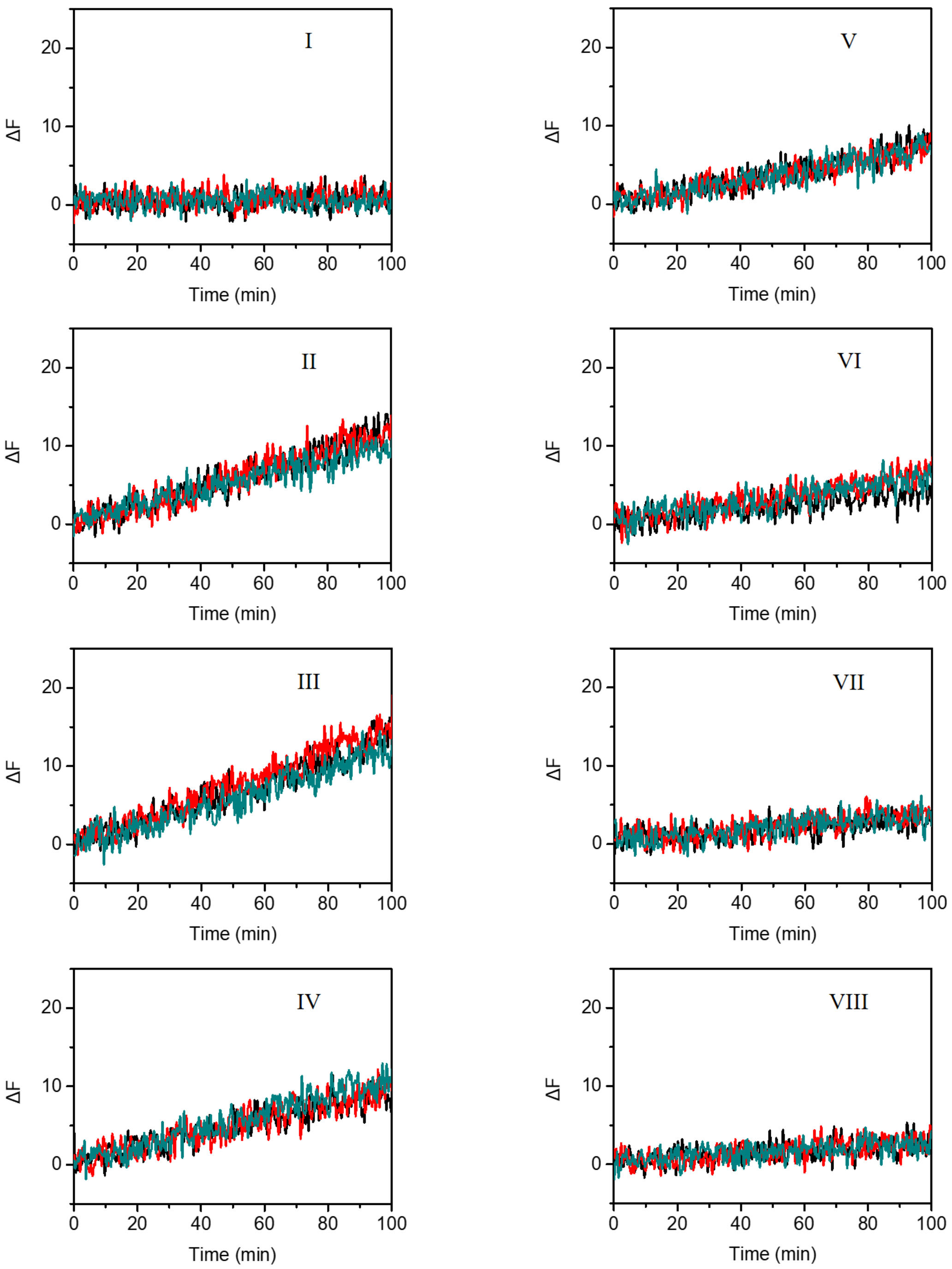

Supplementary Figure 48. Time-dependent catalytic activities of the DNAzyme G_1 generated by the functional coded gene-replication model in the presence of 1.8 μM trigger T_1 at different time intervals: I, 0 h; II, 0.5 h; III, 1.33 h; IV, 6 h; V, 14 h; VI, 26 h; VII, 38 h; VIII, 50 h. Each measurement was repeated three times (N = 3).

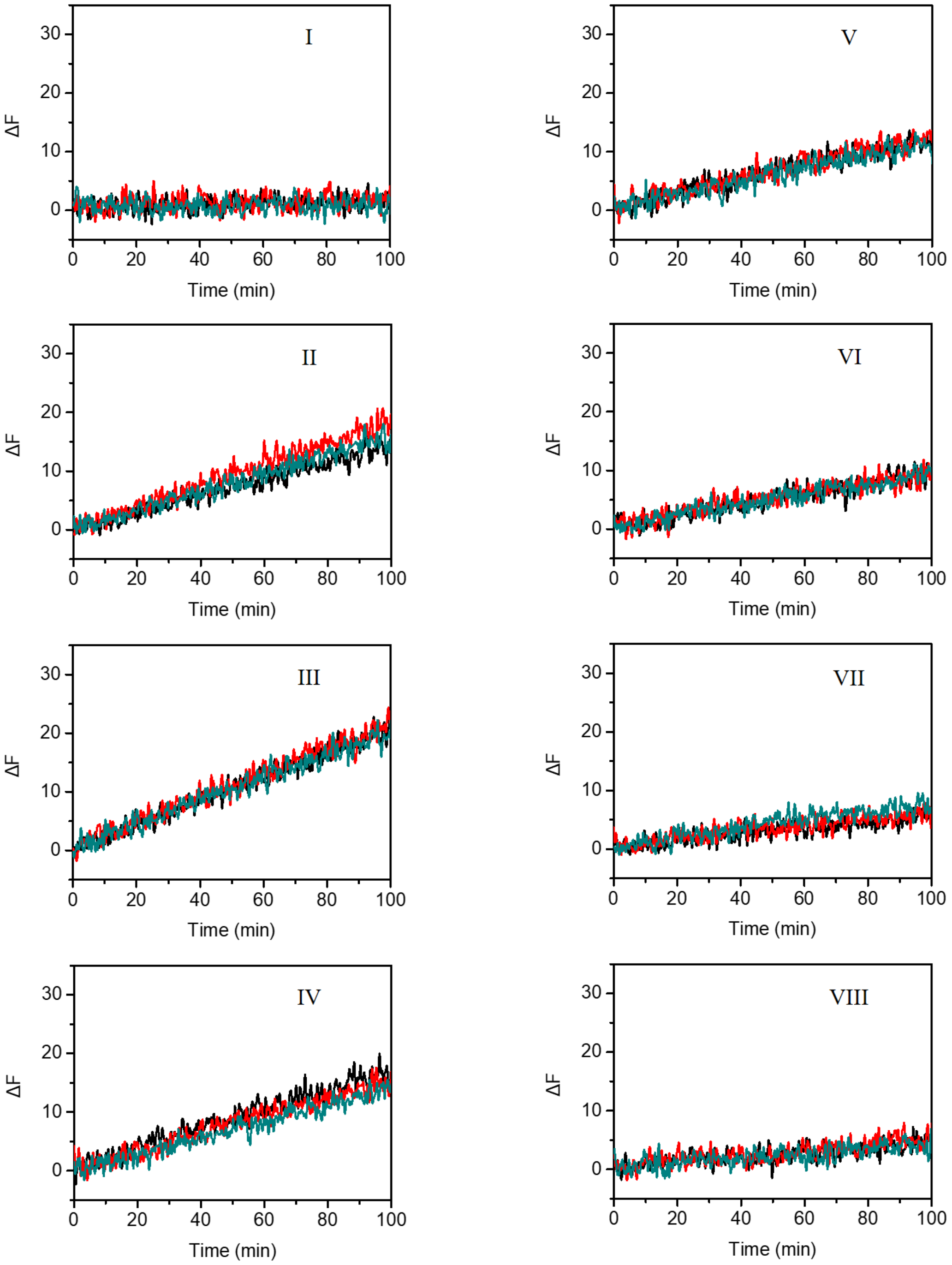

Supplementary Figure 49. Time-dependent catalytic activities of the DNAzyme G_2 generated by the functional coded gene-replication model in the presence of 1.8 μM trigger T_1 at different time intervals: I, 0 h; II, 0.5 h; III, 1.33 h; IV, 6 h; V, 14 h; VI, 26 h; VII, 38 h; VIII, 50 h. Each measurement was repeated three times (N = 3).

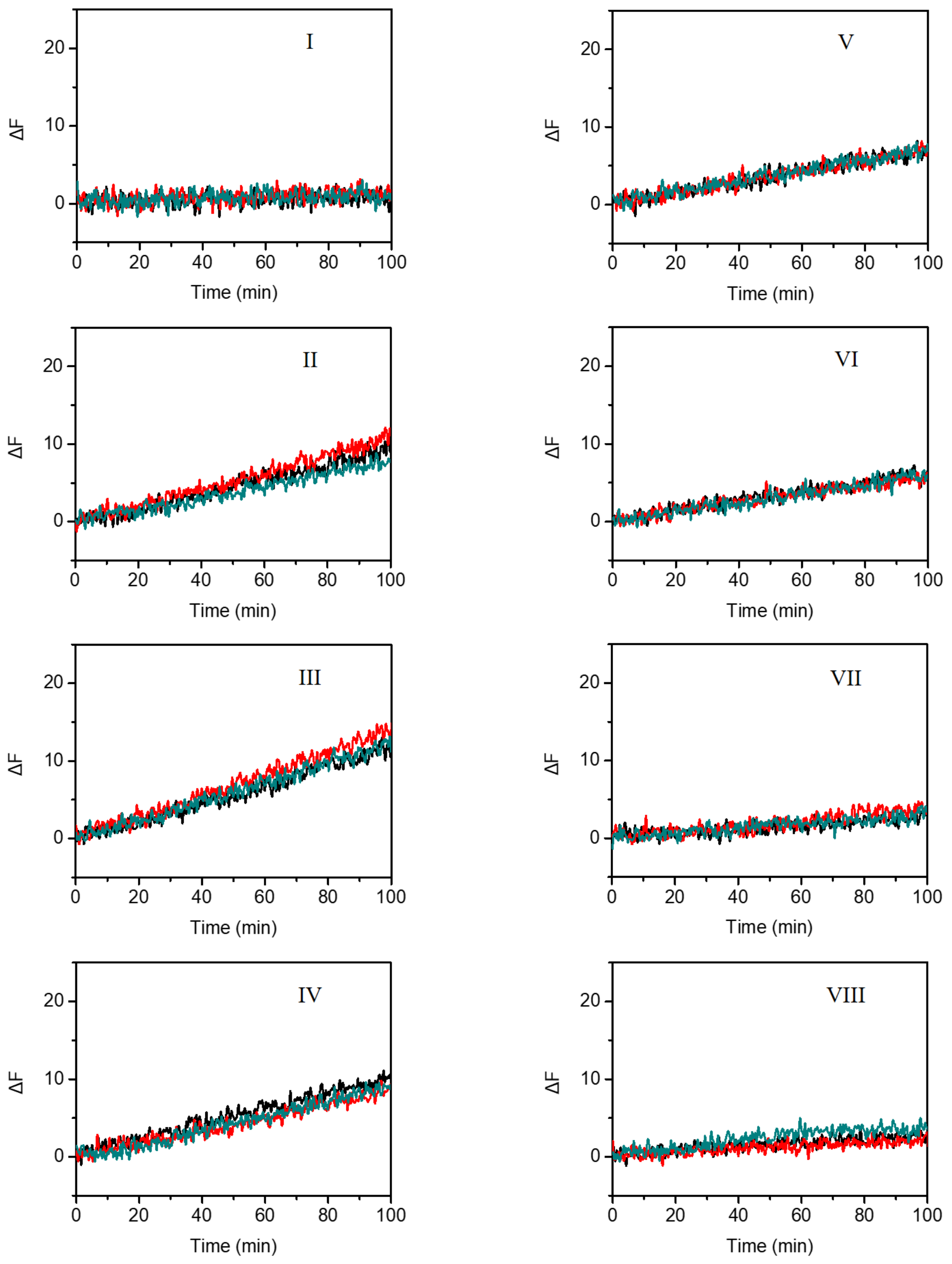

Supplementary Figure 50. Time-dependent catalytic activities of the DNAzyme G_3 generated by the functional coded gene-replication model in the presence of 1.8 μM trigger T_1 at different time intervals: I, 0 h; II, 0.5 h; III, 1.33 h; IV, 6 h; V, 14 h; VI, 26 h; VII, 38 h; VIII, 50 h. Each measurement was repeated three times (N = 3).

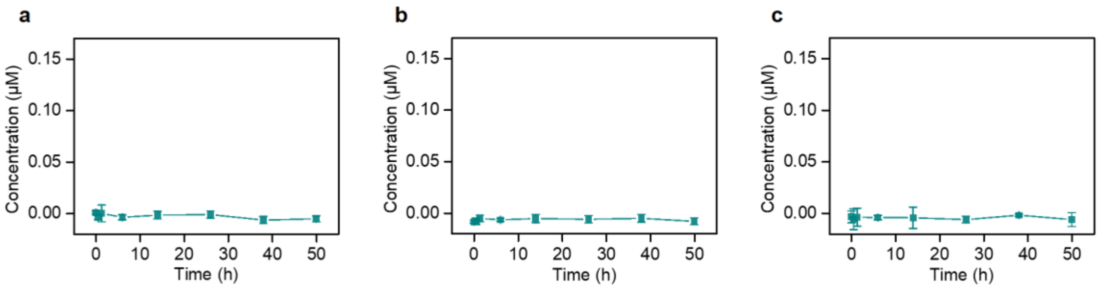

Supplementary Figure 51. Transient concentration changes of the DNAzyme model genes generated by the functional coded gene-replication model excluding the module III in the presence of 3.6 μM T_1: a, DNAzyme G_1; b, DNAzyme G_2; c, DNAzyme G_3. Error bars were derived from N = 3 experiments.

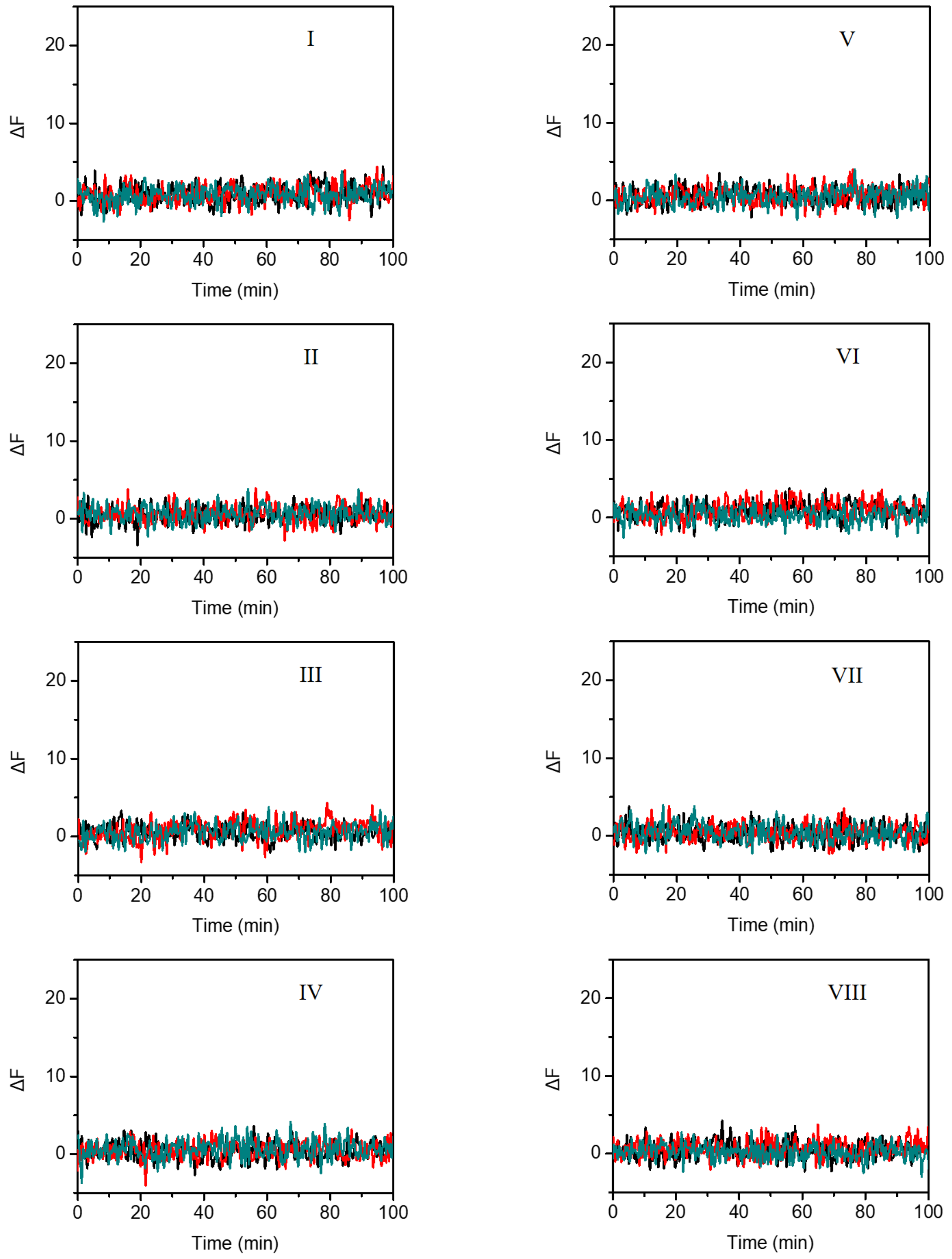

Supplementary Figure 52. Time-dependent catalytic activities of the DNAzyme G_1 generated by the functional coded gene-replication model excluding the module III in the presence of 3.6 μM T_1 at different time intervals: I, 0 h; II, 0.5 h; III, 1.33 h; IV, 6 h; V, 14 h; VI, 26 h; VII, 38 h; VIII, 50 h. Each measurement was repeated three times (N = 3).

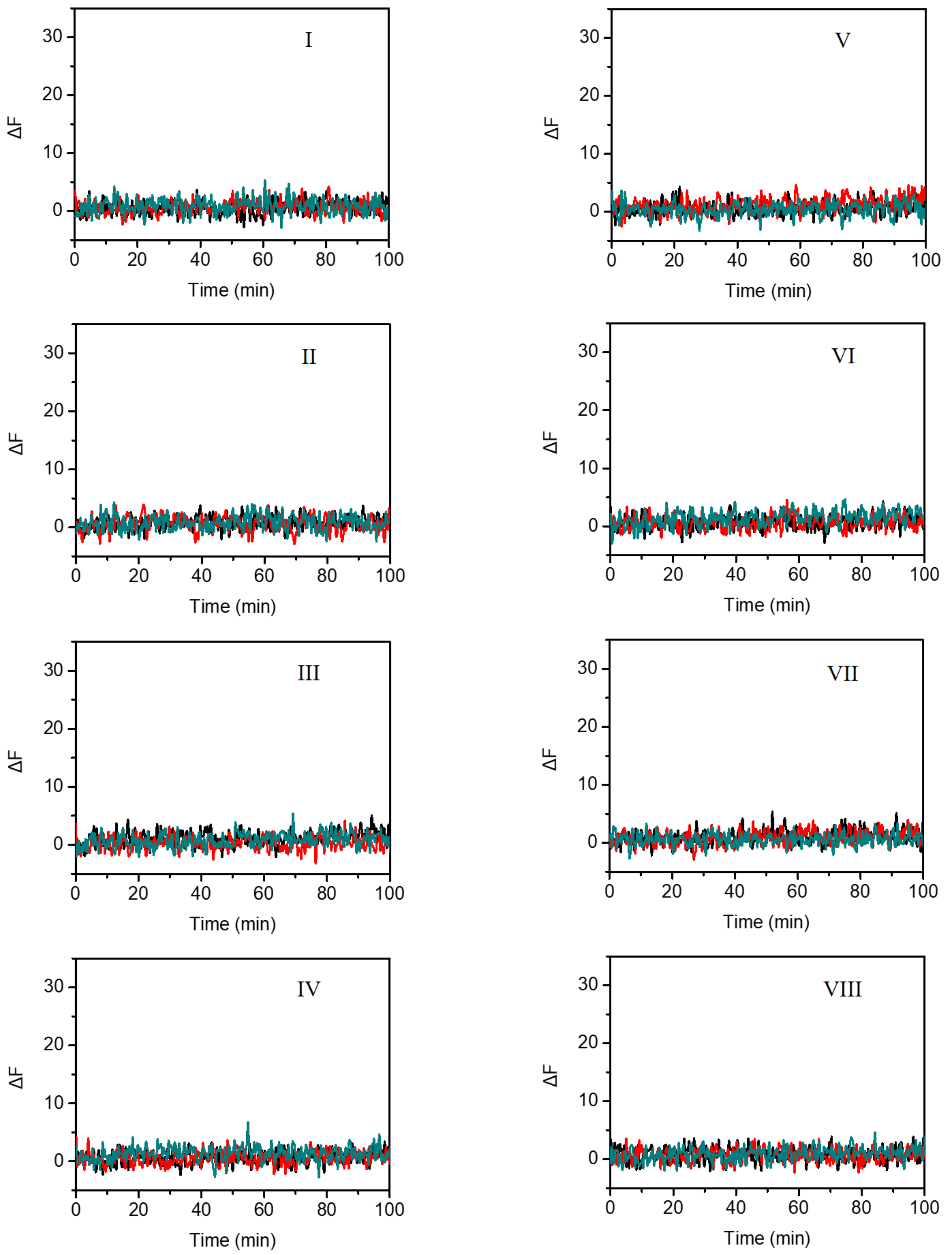

Supplementary Figure 53. Time-dependent catalytic activities of the DNAzyme G_2 generated by the functional coded gene-replication model excluding the module III in the presence of 3.6 µM T_1 at different time intervals: I, 0 h; II, 0.5 h; III, 1.33 h; IV, 6 h; V, 14 h; VI, 26 h; VII, 38 h; VIII, 50 h. Each measurement was repeated three times (N = 3).

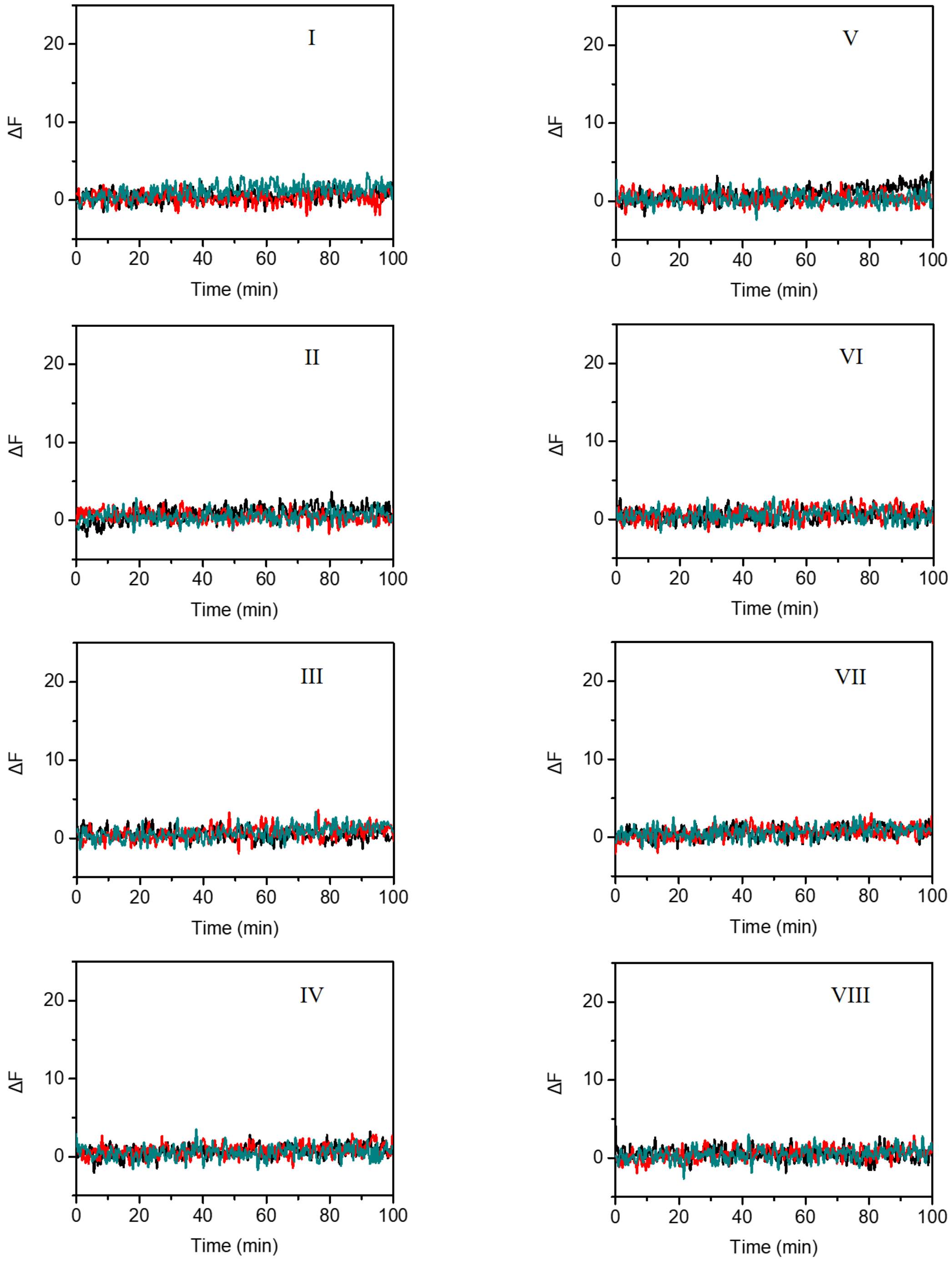

Supplementary Figure 54. Time-dependent catalytic activities of the DNAzyme G_3 generated by the functional coded gene-replication model excluding the module III in the presence of 3.6 μM T_1 at different time intervals: I, 0 h; II, 0.5 h; III, 1.33 h; IV, 6 h; V, 14 h; VI, 26 h; VII, 38 h; VIII, 50 h. Each measurement was repeated three times (N = 3).

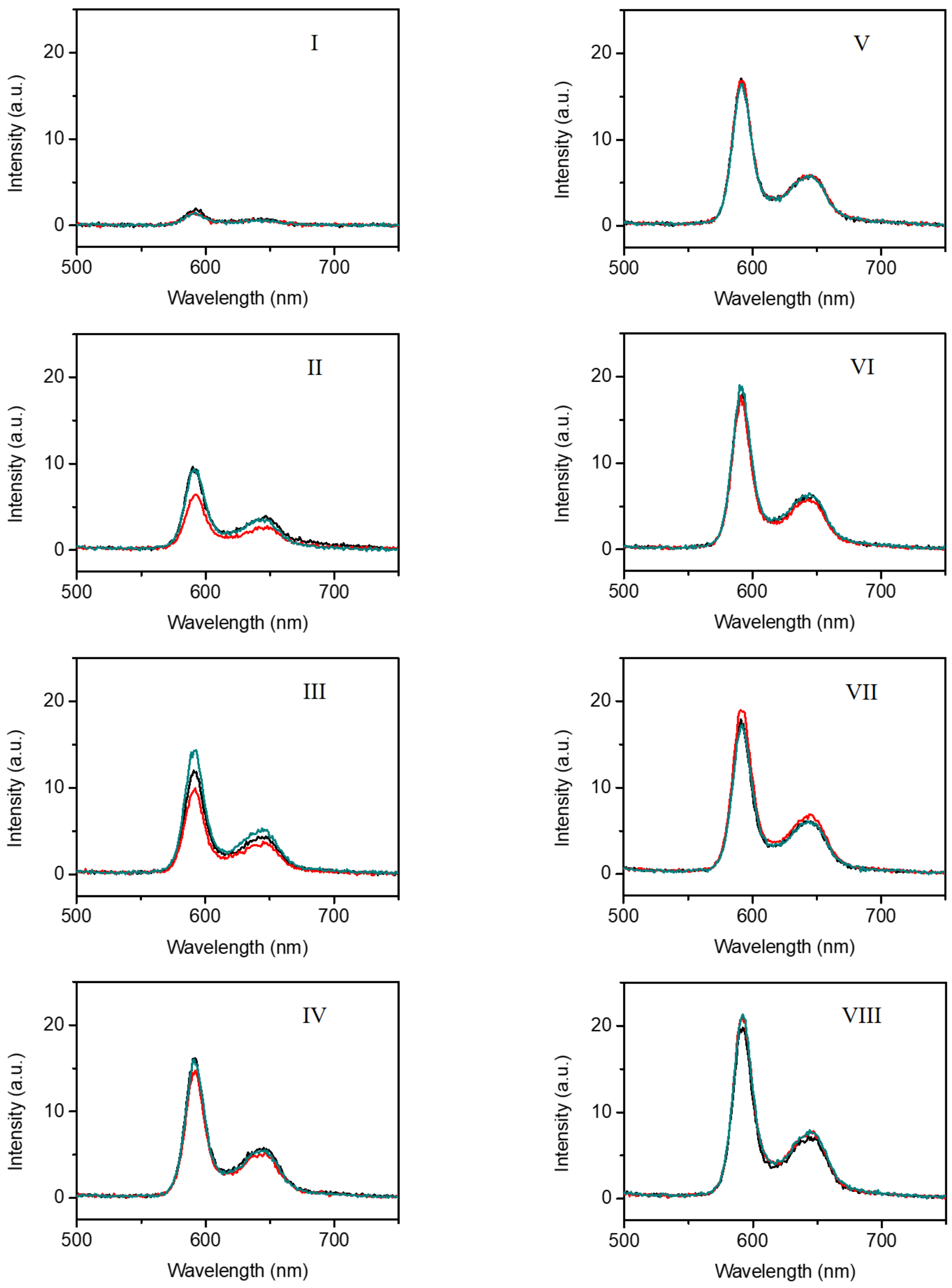

Supplementary Figure 55. The fluorescence spectra of the Zn(II)-PPIX/G-quadruplex wires generated by the RCA induced by the P strand from the three-layer cascade in the presence of 3.6 μM T_1 at different time intervals: I, 0 h; II, 3 h; III, 6 h; IV, 9 h; V, 15 h; VI, 21 h; VII, 30 h; VIII, 42 h. Each of the fluorescence spectra of the respective system shows the spectra of three different measurements (N = 3).

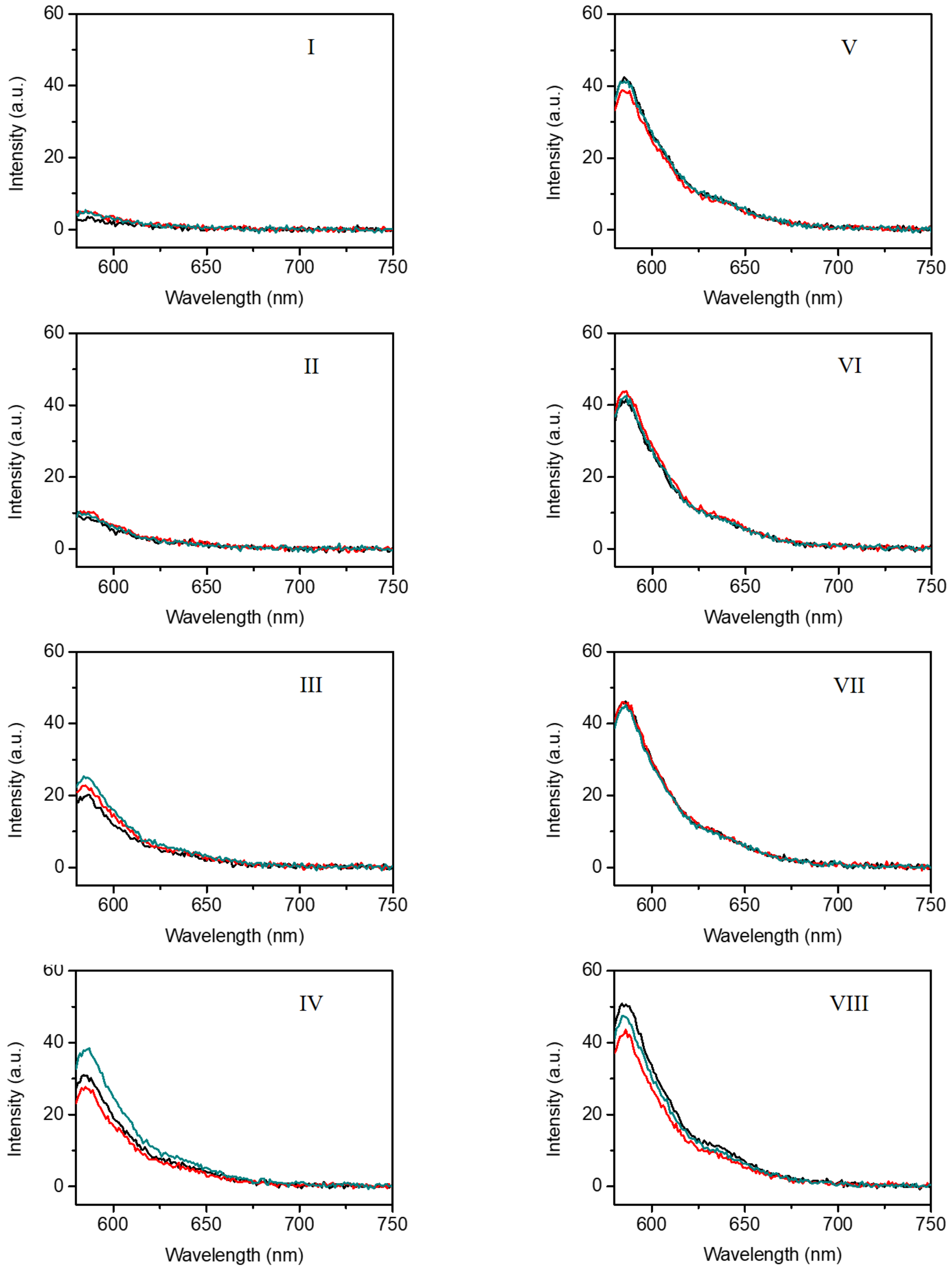

Supplementary Figure 56. The fluorescence spectra of the product Resorufin catalyzed by the hemin/G-quadruplex wires generated by the RCA induced by the P strand from the three-layer cascade in the presence of 3.6 μM T_1 at different time intervals: I, 0 h; II, 3 h; III, 6 h; IV, 9 h; V, 15 h; VI, 21 h; VII, 30 h; VIII, 42 h. Each of the fluorescence spectra of the respective system shows the spectra of three different measurements (N = 3).

Probing the transient cascades in biological environments

The experiments described in the study were performed in pure buffer solutions. It is important, however, for future applications of such networks in biological environments, to examine the feasibility to trigger and operate such nucleic acid components in native environments. Towards this goal, we examined the feasibility to trigger the operation of the single layer, double layer, and three-layer cascade in MCF-10A epithelial breast cell lysate, and MDA-MB-231 breast cancer cell lysate. The results of these experiments are displayed in the following Supplementary Figures. The results allow us to make some general conclusions: (i) The transient cascades operate successfully in the native cell lysates media. (ii) The amplitudes of the transient cascades are lower than in the pure buffer solutions. (iii) The kinetics of the transient cascades are influenced by the reaction media and the transient recovery of the parent reaction moduli is slower. (iv) The successful operation of the DNAzyme cascades in native lysates indicates sufficient stabilities against possible nuclease degradation process on timescales of 20-50 hours. We note, however, that the detailed evaluation of such transient systems in native environments, is far beyond the scope of the present study, and future efforts towards such experiments are certainly an interesting path to follow.

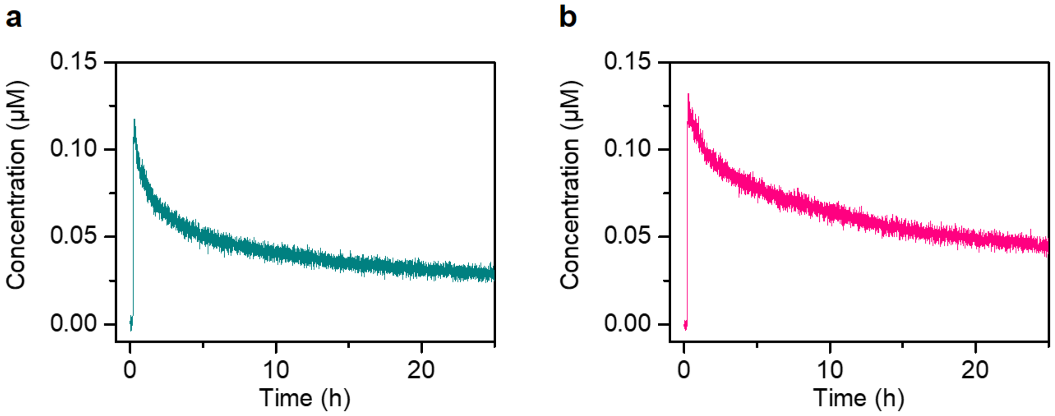

Supplementary Figure 57. Triggered transient formation and depletion of the DNAzyme T_1/A with a concentration of 0.4 μM trigger T_1 in: a, MCF-10A epithelial breast cell lysate (1500 lysed cells for 0.15 mL reaction system). **b,** MDA-MB-231 breast cancer cell lysate (1500 lysed cells for 0.15 mL reaction system).

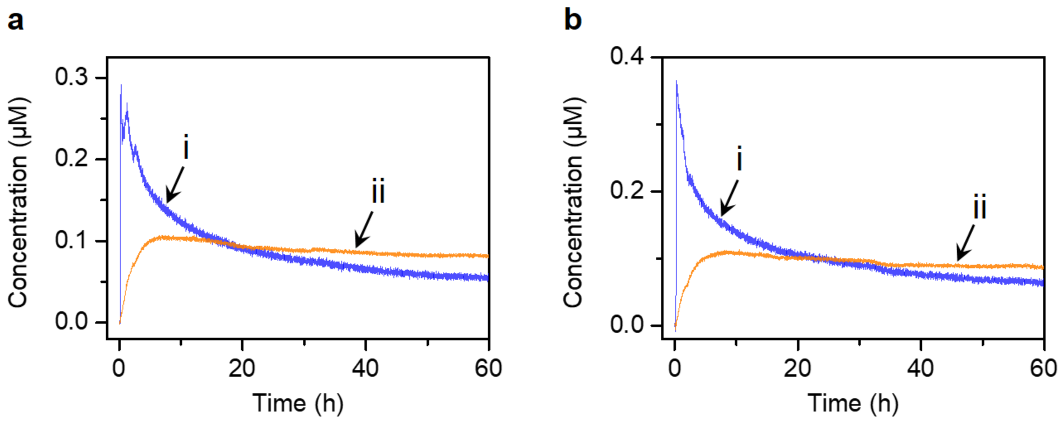

Supplementary Figure 58. Triggered operation of the two-layer transient DNAzyme cascade (module I + module II) shown in Figure 2a in the cell lysates. a, Transient concentration changes corresponding to DNAzyme T_1/A (i) and DNAzyme T_2/B (ii) in MCF-10A epithelial breast cell lysate (1500 lysed cells for 0.15 mL reaction system) in the presence of 1.8 μM trigger T_1. **b,** Transient concentration changes corresponding to DNAzyme T_1/A (i) and DNAzyme T_2/B (ii) in MDA-MB-231 breast cancer cell lysate (1500 lysed cells for 0.15 mL reaction system) in the presence of 1.8 μM trigger T_1.

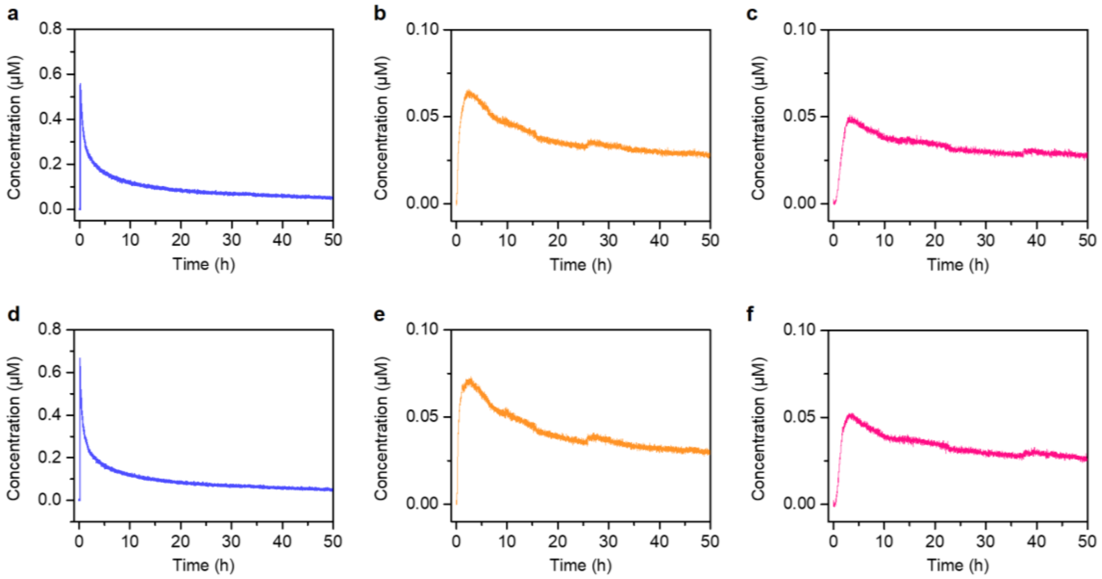

Supplementary Figure 59. Triggered operation of the three-layer transient DNAzyme cascade (module I + module II + module III) shown in Figure 3a in the cell lysates. a-c, Transient concentration changes corresponding to DNAzyme T_1/A (**a**), DNAzyme T_2/B (**b**) and DNAzyme T_3/C (**c**) in MCF-10A epithelial breast cell lysate (1500 lysed cells for 0.15 mL reaction system) in the presence of 3.6 µM trigger T_1. **d-f,** Transient concentration changes corresponding to DNAzyme T_1/A (**d**), DNAzyme T_2/B (**e**) and DNAzyme T_3/C (**f**) in MDA-MB-231 breast cancer cell lysate (1500 lysed cells for 0.15 mL reaction system) in the presence of 3.6 µM trigger T_1.

Activities of the Mg^{2+}-ion-dependent DNAzyme in cell lysates

As the issue of operating the different DNAzyme layered cascades in cell lysates was addressed. We examined the possibility to operate the DNAzyme by physiological concentration of Mg^{2+} ions in native fluids. The reported values of free Mg^{2+} ions in cells is in the range of 0.5-1.5 mM.[1-3] Accordingly, the different cell lysates were subjected to Mg^{2+} ions, 1 mM and 2 mM, and the results of the activities of the DNAzyme are presented in Supplementary Figure 59 and compared to the activities of the DNAzyme in the pure buffer solution with 1 mM and 2 mM Mg^{2+} ions. The results demonstrate that the cell lysates at cellular concentrations of Mg^{2+} allow the operation of the DNAzyme.

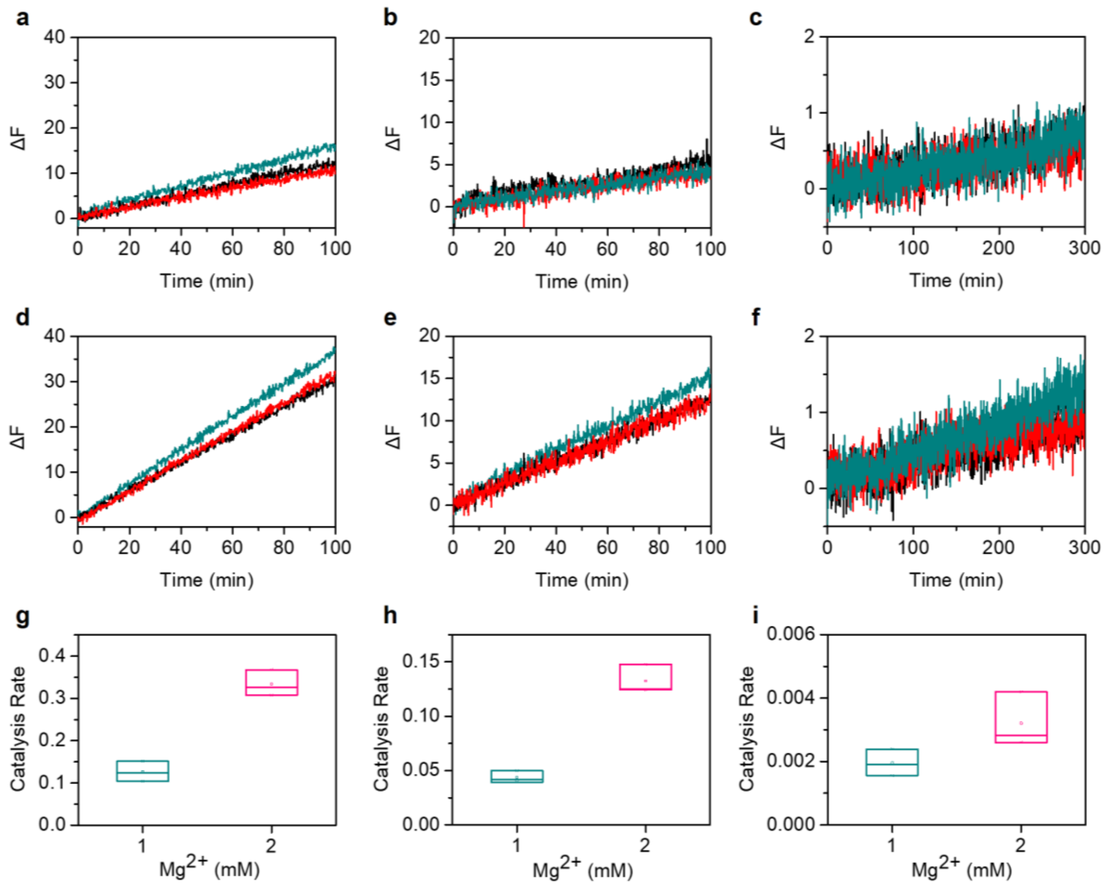

Supplementary Figure 60. Activities of the Mg^{2+}-ion-dependent DNAzyme in cell lysates and pure buffer with added Mg^{2+} ions. a-f, Time-dependent catalytic activities of the Mg^{2+}-ion-dependent DNAzyme (D_1/D_2) with the substrate S_4 in MCF-10A epithelial breast cell lysate (150000 lysed cells for 0.15 mL reaction system, (**a**) and (**d**)), MDA-MB-231 breast cancer cell lysate (150000 lysed cells for 0.15 mL reaction system, (**b**) and (**e**)) and pure buffer, (**c**) and (**f**), with added Mg^{2+} ions of 1 mM, (**a**), (**b**) and (**c**), and 2 mM, (**d**), (**e**) and (**f**), respectively. **g-i,** Activities of the Mg^{2+}-ion-dependent DNAzyme (D_1/D_2) with the substrate S_4 in MCF-10A epithelial breast cell lysate (150000 lysed cells for 0.15 mL reaction system, (**g**)), MDA-MB-231 breast cancer cell lysate (150000 lysed cells for 0.15 mL reaction system, (**h**)) and pure buffer, (**i**), with added Mg^{2+} ions of 1 mM and 2 mM, respectively (N = 3).

Supplementary Table 1. Rate constants derived from the computational simulation of the dissipative system shown in Figure 1.

k_1	2.88123 μM^{-1} min^{-1}	k_4	99 min^{-1}	k_{-6}	0.07223 μM^{-1} min^{-1}	
k_{-1}	3.54853 μM^{-1} min^{-1}	k_{-4}	0.0001 μM^{-1} min^{-1}	k_7	17.82 μM^{-1} min^{-1}	17.815 μM^{-1} min^{-1}
k_2	6 μM^{-1} min^{-1}	k_5	0.002 min^{-1}	k_{-7}	0.036 min^{-1}	0.0367 min^{-1}
k_{-2}	10 min^{-1}	k_{-5}	5.76616 μM^{-1} min^{-1}			
k_3	0.85 min^{-1}	k_6	6 μM^{-1} min^{-1}			

Supplementary Table 2. Rate constants derived from the computational simulation of the dissipative system shown in Supplementary Figure 7.

k_8	0.1222 μM^{-1} min^{-1}	k_{11}	89 min^{-1}	k_{-13}	0.03724 μM^{-1} min^{-1}	
k_{-8}	2.01813 μM^{-1} min^{-1}	k_{-11}	0.001 μM^{-1} min^{-1}	k_{14}	10.32 μM^{-1} min^{-1}	10.3 μM^{-1} min^{-1}
k_9	6 μM^{-1} min^{-1}	k_{12}	0.002 min^{-1}	k_{-14}	0.0098 min^{-1}	0.01 min^{-1}
k_{-9}	10 min^{-1}	k_{-12}	3.14923 μM^{-1} min^{-1}			
k_{10}	0.85 min^{-1}	k_{13}	9 μM^{-1} min^{-1}			

Supplementary Table 3. Rate constants derived from the computational simulation of the dissipative system shown in Supplementary Figure 12.

k_{15}	0.3287 μM^{-1} min^{-1}	k_{18}	50 min^{-1}	k_{-20}	0.01869 μM^{-1} min^{-1}	
k_{-15}	0.81678 μM^{-1} min^{-1}	k_{-18}	10 μM^{-1} min^{-1}	k_{21}	37.6 μM^{-1} min^{-1}	37.477 μM^{-1} min^{-1}
k_{16}	3 μM^{-1} min^{-1}	k_{19}	0.05min^{-1}	k_{-21}	0.08 min^{-1}	0.0936 min^{-1}
k_{-16}	43.56105 min^{-1}	k_{-19}	34 μM^{-1} min^{-1}			
k_{17}	0.85 min^{-1}	k_{20}	7.27093 μM^{-1} min^{-1}			

Supplementary Table 4. Rate constants derived from the computational simulation of the two-layer cascaded dissipative system shown in Figure 2.

k_{22}	0.62548 μM^{-1} min^{-1}	k_{-27}	5 μM^{-1} min^{-1}	k_{33}	0.02241 min^{-1}
k_{-22}	2.03843 μM^{-1} min^{-1}	k_{28}	17 μM^{-1} min^{-1}	k_{-33}	10 μM^{-1} min^{-1}
k_{23}	4.78506 μM^{-1} min^{-1}	k_{-28}	0.0006 min^{-1}	k_{34}	10 μM^{-1} min^{-1}
k_{-23}	7.94903 min^{-1}	k_{29}	0.99928 μM^{-1} min^{-1}	k_{-34}	0.01533 μM^{-1} min^{-1}
k_{24}	0.84999 min^{-1}	k_{-29}	0.06009 μM^{-1} min^{-1}	k_{35}	10.3 μM^{-1} min^{-1}
k_{25}	9.99959 min^{-1}	k_{30}	9.92523 μM^{-1} min^{-1}	k_{-35}	0.00073 min^{-1}
k_{-25}	0.24113 μM^{-1} min^{-1}	k_{-30}	5.13428 min^{-1}	k_{36}	0.04621 μM^{-1} min^{-1}
k_{26}	0.10858 min^{-1}	k_{31}	0.85 min^{-1}	k_{-36}	10 μM^{-1} min^{-1}
k_{-26}	59.99997 μM^{-1} min^{-1}	k_{32}	2.0409 min^{-1}		
k_{27}	6.4443 μM^{-1} min^{-1}	k_{-32}	5 μM^{-1} min^{-1}		

Supplementary Table 5. Rate constants derived from the computational simulation of the two-layer cascaded dissipative system shown in Supplementary Figure 24.

k_{37}	0.15009 μM^{-1} min^{-1}	k_{-42}	4.98457 μM^{-1} min^{-1}	k_{48}	0.65200 min^{-1}
k_{-37}	1.30309 μM^{-1} min^{-1}	k_{43}	10.20165 μM^{-1} min^{-1}	k_{-48}	10.55877 μM^{-1} min^{-1}
k_{38}	5.20377 μM^{-1} min^{-1}	k_{-43}	0.00058 min^{-1}	k_{49}	9.99999 μM^{-1} min^{-1}
k_{-38}	9.99994 min^{-1}	k_{44}	2.99996 μM^{-1} min^{-1}	k_{-49}	4.92511 μM^{-1} min^{-1}
k_{39}	0.85 min^{-1}	k_{-44}	1.4794 μM^{-1} min^{-1}	k_{50}	37.00293 μM^{-1} min^{-1}
k_{40}	10 min^{-1}	k_{45}	1.6219 μM^{-1} min^{-1}	k_{-50}	0.00063 min^{-1}
k_{-40}	0.96936 μM^{-1} min^{-1}	k_{-45}	1.4847 min^{-1}	k_{51}	0.44443 μM^{-1} min^{-1}
k_{41}	0.1 min^{-1}	k_{46}	0.14041 min^{-1}	k_{-51}	10 μM^{-1} min^{-1}
k_{-41}	29.7033 μM^{-1} min^{-1}	k_{47}	0.11525 min^{-1}		
k_{42}	10 μM^{-1} min^{-1}	k_{-47}	4.99999 μM^{-1} min^{-1}		

Supplementary Table 6. Rate constants derived from the computational simulation of the three-layer cascaded dissipative system shown in Figure 3.

k_{52}	0.12136 μM^{-1} min^{-1}	k_{60}	1.44588 μM^{-1} min^{-1}	k_{68}	1.50647 min^{-1}
k_{-52}	4.58105 μM^{-1} min^{-1}	k_{-60}	9.67933 min^{-1}	k_{69}	9.80017 min^{-1}
k_{53}	3.01591 μM^{-1} min^{-1}	k_{61}	0.84997 min^{-1}	k_{-69}	1.45672 μM^{-1} min^{-1}
k_{-53}	9.54809 min^{-1}	k_{62}	9.99813 min^{-1}	k_{70}	0.19146 min^{-1}
k_{54}	0.85 min^{-1}	k_{-62}	0.51937 μM^{-1} min^{-1}	k_{-70}	0.01814 μM^{-1} min^{-1}
k_{55}	10 min^{-1}	k_{63}	0.08273 min^{-1}	k_{71}	0.2515 μM^{-1} min^{-1}
k_{-55}	2.43848 μM^{-1} min^{-1}	k_{-63}	27.4869 μM^{-1} min^{-1}	k_{-71}	3.01338 μM^{-1} min^{-1}
k_{56}	0.31673 min^{-1}	k_{64}	8.32421 μM^{-1} min^{-1}	k_{72}	6.54718 μM^{-1} min^{-1}
k_{-56}	43.23015 μM^{-1} min^{-1}	k_{-64}	0.01506 μM^{-1} min^{-1}	k_{-72}	18.69906 min^{-1}
k_{57}	10 μM^{-1} min^{-1}	k_{65}	10.3 μM^{-1} min^{-1}	k_{73}	5.89179 μM^{-1} min^{-1}
k_{-57}	5 μM^{-1} min^{-1}	k_{-65}	0.00055 min^{-1}	k_{-73}	1.3546 μM^{-1} min^{-1}
k_{58}	17 μM^{-1} min^{-1}	k_{66}	0.34573 μM^{-1} min^{-1}	k_{74}	37.05306 μM^{-1} min^{-1}
k_{-58}	0.00055 min^{-1}	k_{-66}	9.99985 μM^{-1} min^{-1}	k_{-74}	0.00999 μM^{-1} min^{-1}
k_{59}	0.15783 μM^{-1} min^{-1}	k_{67}	0.18575 μM^{-1} min^{-1}	k_{75}	2.55649 μM^{-1} min^{-1}
k_{-59}	0.09845 μM^{-1} min^{-1}	k_{-67}	9.99991 min^{-1}	k_{-75}	9.83409 μM^{-1} min^{-1}

Supplementary Table 7. Sequences of oligonucleotides.

A	Cy5-CATCTGGATGGAATCTGACCTTCACCCATGTCTGTTG
A'	TCGTTAAGATTCTATCCAGATG-BHQ2
H_1	ACAGACTCCGTTCAAACTTAAACAACAGT **rA** GGAGAATCAAGTAGTTTGAAT GGAGTCTGAC
T_1	GATTCTCAGCGATAAGGTCAGATTCCATTCAAACTAC
H_{1c}	ACAGACTCCGTTCAAACTTAAACAACAGTAGGAGAATCAAGTAGTTTGAAT GGAGTCTGAC
B	FAM-TGAGAATCGGTTTGTCCTCCGTTCACCCATGTAGTCTG
B'	TTGGATGGACAGACCGGTTCTC-BHQ1
H_2	CTAAGGCAAGCTGATTGAGTAAACAGACTT **rA** GGAATCACAAGCCTCAATCA GTTTGCCTTC
T_2	GTGATTCAGCGATAACGGAGGGCAAACTGATTGAGGC
H_{2c}	CTAAGGCAAGCTGATTGAGTAAACAGACTTAGGAATCACAAGCCTCAATCA GTTTGCCTTC
C	CY3-GTCAAGACTCAGTGACCCTTAGCACCCATGTACTGTC
C'	GAGGGAGTTGCTGGGTCTTGAC-BHQ2
H_3	GGTCGTTAGGCTTCCATTAAAGACAGTT **rA** GGAAAGAGAAGTATGGAAGCTT AATGACCC
T_3	CTCTTTCAGCGATCTAAGGGTCATTGAGTTTCCATAC
H_{3c}	GGTCGTTAGGCTTCCATTAAAGACAGTTAGGAAAGAGAAGTATGGAAGCTT AATGACCC
S	CTCAATCAGTTTGAACGGAGTCTGTTATCG
M	ATGGAAACTCAATCAGCTTGCCTTAGATCGC
W	CAGTAAGAATGGAAGCCTAACGACCGATCACA
P	TGTGATCGGTTGTCTCTCTATTCTTACTG
J_1	AGAGTAACATGGGTGTAACCTGGTTAATCGCTGAGCAGGCTGAGGGTCAAGA CTCAGTG
J_2	TGACGAACATGGGTGTAACCTGGTTAATCGCTGAGCAGGCTGAGGGTCAAGA CTCAGTG
J_3	AGCTAAACATGGGTGTAACCTGGTTAATCGCTGAGGACGCTGAGGGTCAAGA CTCAGTG
G_1	TCAGCCTGCTCAGCGATTAACCAGGTTACACCCATGTTACTCT
G_2	TCAGCCTGCTCAGCGATTAACCAGGTTACACCCATGTTCGTCA
G_3	TCAGCGTCCTCAGCGATTAACCAGGTTACACCCATGTTTAGCT
S_1	Cy5-AGAGTAT **rA** GGAGCAG-BHQ2
S_2	ROX-TGACGAT **rA** GGAGCAG-BHQ2
S_3	FAM-AGCTAAT **rA** GGAGGAC-BHQ1
T_R	*Phos*-GAATAGAACCCACCCACCCACCCAAAACCCACCCACCCACCCAAAACC CACCCACCCACCCCAGTAA
C_L	TGGGTTCTATTCTTACTGGGGTGG
D_1	CATCTGGATGGAATCTGACCTTTAACCACCCATGTTCCTGA
D_2	CTGTTCAGCGATGTTAAAGGTCAGATTCCATTCAAACTAC

S_4	FAM-TCAGGAT **rA** GGAACAG-IBRQ

Supplementary References

1. Murphy, E., Freudenrich, C. C., Levy, L. A. & London, R. E. Monitoring cytosolic free magnesium in cultured chicken heart cells by use of the fluorescent indicator Furaptra. *Proc. Natl. Acad. Sci. U. S. A.*, **86**, 2981–2984 (1989).
2. Murphy, E., Steenbergen, C., Levy, L. A., Raju, B. & London, R. E. Cytosolic free magnesium levels in ischemic rat heart. *J. Biol. Chem.*, **264**, 5622–5627 (1989).
3. Lennard, R. & Singh, J. Secretagogue-evoked changes in intracellular free magnesium concentrations in rat pancreatic acinar cells. *J. Physiol.*, **435**, 483–492 (1991).
